# The effect of neuroscientific evidence on sentencing depends on how one conceives of reasons for incarceration

**DOI:** 10.1371/journal.pone.0276237

**Published:** 2022-11-02

**Authors:** Annalise Perricone, Arielle Baskin-Sommers, Woo-kyoung Ahn

**Affiliations:** Department of Psychology, Yale University, New Haven, Connecticut, United States of America; University of Padova, ITALY

## Abstract

Neuroscientific evidence is increasingly utilized in criminal legal proceedings, prompting discussions about how such evidence might influence legal decisions. The effect of neuroscientific testimony on legal decisions remains uncertain, with some studies finding no effect, others reporting that neuroscience has a mitigating impact, and some indicating neuroscience evidence has an aggravating effect. The present study attempts to explain these divergent findings by showing that the effect of neuroscience evidence on sentencing interacts with beliefs about the goals of the criminal legal system. Using a between-subjects design, participants (*N* = 784) were asked to assume different rationales for imprisonment, before receiving neuroscientific evidence about antisocial behavior and its potential relation to the defendant. Participants recommended a sentence for the defendant prior to and after reading the neuroscientific evidence. Participants who were given the rationale of retribution as the primary goal of imprisonment significantly decreased their sentencing recommendations. When the goal of imprisonment was to protect the public from dangerous people, participants provided longer post-testimony sentences. Lastly, when the goal was to rehabilitate wrongdoers, participants also increased sentences from pre to post. Thus, the impact of neuroscientific evidence is not monolithic, but can lead to either mitigated or aggravated sentences by interacting with penal philosophy.

## Introduction

The past several decades have seen a rapid rise in the use of neuroscientific evidence in criminal proceedings. Illustrating this trend, in the United States there were approximately 100 judicial opinions that discussed neuroscientific evidence in 2005, whereas by 2012 there were as many as 300 [1]. In response to this changing legal landscape, scholars and lay people alike have expressed concerns about how the use of neuroscience evidence may affect legal decisions [2–6]. In particular, neuroscientists and legal scholars have warned that neuroscience claims [7] used in criminal proceedings may exaggerate the scientific findings upon which they are based and that such claims may fail to adequately express the limitations of neuroscience [1, 8–10]. Thus fears have been raised that neuroscience may have a special persuasive power, or “seductive allure” that renders even illogical and irrelevant pseudo-scientific explanations convincing [11].

### Review of the effects of neuroscientific evidence

Despite these concerns, the actual effect of neuroscientific testimony on legal decisions remains unclear. For example, it is estimated that only 20% of defendants achieve a favorable outcome after using neuroscience evidence in their defense [1]. Consistent with what has been observed in real-world court settings, empirical findings on the effect of neuroscientific evidence on legal decisions also have been mixed (see [12] for a review; [13]).

Some studies have found that neuroscientific evidence has no effect on hypothetical verdicts and sentences [14–20]. For instance Schweitzer and colleagues [19] examined the effect of neuroscientific testimony on guilty vs. not guilty verdicts and sentencing decisions. Mock jurors received a case, describing a defendant who was charged with murder, along with the evidence from an expert who testified that a brain scan revealed a frontal lobe defect that caused the defendant to be incapable of the intent necessary to be found guilty of murder. Across the four experiments, the neuroscientific testimony failed to impact the mock jurors’ verdicts and sentencing decisions. Similarly, Appelbaum, Scurich and Raad [21] found that when genetic and neurobiological evidence was presented as expert testimony concerning the defendant’s predisposition towards violent and impulsive behavior, the testimony failed to significantly affect lay participants’ prison sentencing decisions.

In contrast, others have found that such evidence can be mitigating [22–26]. Aspinwall and colleagues [22] found that presenting participants with neurobiological evidence about a defendant charged with aggravated battery significantly reduced the suggested sentence. In this study, a sample of U.S. judges read a vignette (described as expert testimony) about a defendant who had been diagnosed with psychopathy. In addition to this vignette, some judges also read expert testimony from a neurobiologist who identified biological mechanisms that can lead to the development of psychopathy. Sentences among the judges who were given the neurobiological evidence were significantly lower than among those given no such evidence.

Finally, some studies reported that neuroscience evidence can be aggravating [27, 28]. For example, Appelbaum and Scurich [27] found that genetic evidence about a defendant lengthened lay participants sentencing recommendations. In this study, the defendant had committed an impulsive homicide, and the genetic evidence was used to explain his tendency towards impulsivity and violence. However, the genetic evidence increased sentences, when this evidence was combined with testimony about the defendant’s history of abuse as a child.

### Proposed explanations for the mixed results

What might explain these divergent effects? There may be multiple reasons, including the type of the crime committed by the defendant, whether the defendant is described as having a psychological disorder (e.g., psychopathy), the type of neuroscience evidence presented, and the comprehensibility of the evidence. Additionally, one reason for the mixed results also may be that the effect of any evidence, including neuroscience, on sentencing interacts with people’s views on the goals of the criminal legal system.

According to penal philosophy, there are three prevailing goals of imprisonment (see [29–31]): the goal of punishing wrongdoers for the sake of retribution, the goal of incapacitating those convicted of crimes and keeping the public safe, and the goal of rehabilitating wrongdoers to help them re-enter society without posing as much risk to others (see the discussion section for other possible penal philosophies). In the United States, the rehabilitation rationale enjoyed some popularity in the 1970s and early 1980s, but by the mid-1980s, the rationales of retribution and incapacitation had come to the forefront [31–33]. Other countries—notably those throughout Scandinavia—use the rehabilitation rationale to design their prison systems [34]. It is possible that the specific rationale guiding incarceration in a given system can cause individuals to consider neuroscientific evidence as either mitigating or aggravating.

To begin, we will discuss the retribution rationale. In a criminal legal context, retribution is the infliction of an aversive consequence on an individual who breaks the law [31]. Specifically, imprisonment punishes by depriving the individual of their physical freedom, psychological well-being, material possessions, and other financial resources. In addition, imprisonment may result in physical harm in the form of violence from correctional officers or other incarcerated individuals [31]. The punishment serves as a form of retribution in proportion to the wrongs committed against another individual and/or society [31]. If people view the goal of incarceration as retribution, neuroscientific explanations for a wrongdoer’s behaviors could mitigate sentencing because people perceive neurobiological and genetic characteristics as inborn and outside the individual’s control [35–37]. As a result, the person also is seen as less blameworthy (e.g., [13, 35, 38, 39]) and, therefore, less deserving of retributive punishment [40–42].

A second rationale for imprisonment is the incapacitation of dangerous or law-breaking people by removing them from society. Incarceration according to this rationale also might require the state to determine an individual’s likelihood of future dangerousness and recidivism [31]. When people consider incapacitation for the sake of public safety as a rationale for imprisonment, they could consider neuroscientific evidence about a defendant to be aggravating because people perceive neurobiological and genetic characteristics to be immutable [35, 36, 43]. If an individual has a biogenetic risk factor for antisocial behavior, people may incorrectly interpret this to mean that antisocial behavior is a fixed trait grounded in their unchanging, biogenetic essence [37, 40–42]. Therefore, the individual will be seen as a more permanent threat to public safety [13].

A third rationale for imprisonment is rehabilitation. Rehabilitation is the process of changing unwanted behaviors by some intervention [31], for instance treatment programs in prison. When people consider rehabilitation as a rationale for imprisonment, they might consider neuroscientific evidence about a defendant to aggravate sentences. As demonstrated in past studies, when people were presented with biological attributions for psychological disorders, they became pessimistic about recovery [44–47] because neurobiological and genetic characteristics are believed to be relatively unchangeable. Therefore, when incarceration is conceived as rehabilitation, neurobiological evidence may make people recommend longer terms of sentencing because they will be pessimistic about the defendant’s likelihood of recovery.

Taking these rationales, we suggest that the mixed or null effects of neuroscience evidence on sentencing were obtained in the past studies because people holding different views may cancel out the effects within the same study [40]. That is, if some participants were thinking about retribution, their sentencing recommendations may have been mitigated with neuroscience evidence, whereas others may have considered public safety or rehabilitation, which may have aggravated their sentences. Alternatively, when someone considers more than one of these rationales simultaneously, they may show little change in their sentencing decisions with neuroscientific evidence, thus leading to null findings.

### Overview of the present study

The present study aims to provide the first test of how different rationales for imprisonment affect people’s use of neuroscientific evidence about antisocial behavior and its potential relation to the defendant when recommending a sentence. In order to do this, we instructed participants to consider a hypothetical country which holds one of the different rationales for imprisonment (i.e., retribution, safety for the public, rehabilitation), before they received neuroscientific testimony about a defendant. That is, we devised manipulations to explicitly describe different penal philosophies, thereby removing the ambiguity around which concerns are being activated with neuroscientific evidence. Before and after participants received the neuroscientific evidence, they were asked to recommend a sentence length for the defendant. We hypothesized that neuroscience explanations for the defendant’s crime would increase the prison sentence in the country whose penal philosophy is based on rehabilitation or protecting the public from the danger, whereas it would decrease the prison sentence in the country whose penal philosophy is based on retribution. In addition to the three conditions where different goals of incarceration were explicated, there also was a control condition which did not specify any rationale, and given the mixed results in the past when different philosophies are left for participants to weigh in differently, we did not have any specific hypothesis about the control condition.

## Materials and methods

### Participants and recruitment

Participants were recruited online, using Amazon mTurk Toolkit, a platform developed by Cloud Research. Eight hundred and two U.S. adults completed the study. In accordance with much of the previous work in this area (e.g., [14, 24, 27]), the present study used lay samples to render sentencing recommendations, although in the real world sentencing typically falls to judges.

Participants were excluded if they failed to demonstrate comprehension of the study vignettes (i.e., if they failed an attention check twice), or if they failed to meet the minimum educational criteria (i.e., some college-level coursework or an associate degree), which were used to ensure that participants possessed the reading abilities required to comprehend the study vignettes, including a passage about neuroscience.

### Design, stimuli, and procedures

**Fig 1:**
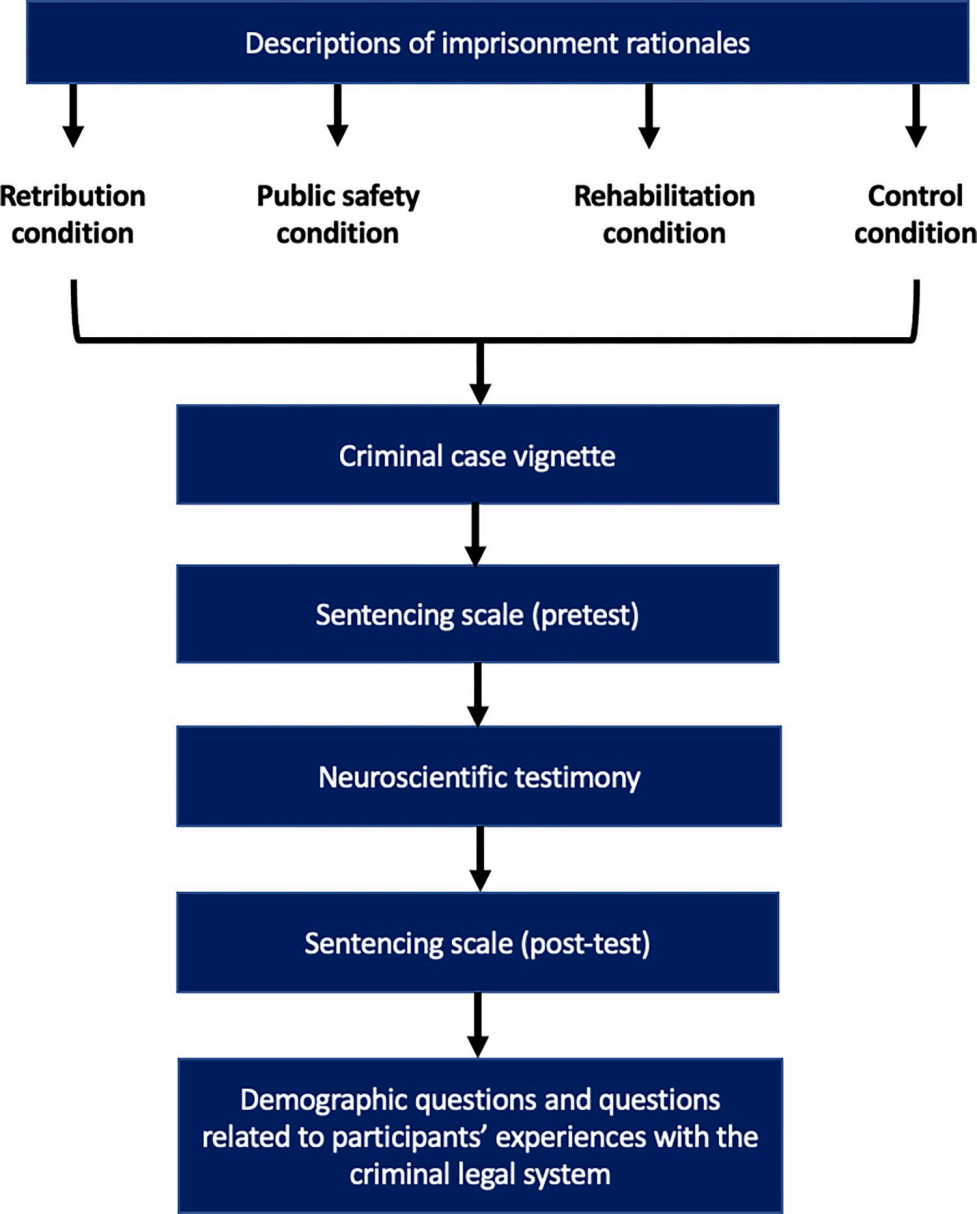
Study procedures.

This study is pre-registered at Open Science Foundation (see https://osf.io/c5vr4). The Yale University Institutional Review Board approved the study. All study procedures (see Fig 1) were administered online, using Qualtrics.com survey software. Prior to beginning the study procedures, participants were provided with an online informed consent form. This form described basic information about the study and only those participants who checked the form’s box labeled, “I have read the above information and agree to participate in the study” were permitted to proceed. After providing consent, participants were randomly assigned to a control condition or to one of three experimental conditions.

Participants assigned to one of the experimental conditions first read a brief, condition-specific vignette describing the primary rationale for incarceration in an unidentified country. The three “country” vignettes described, respectively, the rationale 1) to exact retribution on wrongdoers, 2) to keep the public safe from dangerous law-breakers, and 3) to rehabilitate those convicted of crimes. In these vignettes, participants were explicitly instructed at the end that length of sentence in this country should be based on the stated rationale for imprisonment (see Table 1 for the vignettes). All participants in the experimental conditions then received an attention check on the information they had just received about the rationale for imprisonment in a given country (e.g., “the length of sentence should be based on the time needed to hold people accountable and punish them for their past crimes” vs. “the length of sentence should NOT be based on the time needed to hold people accountable and punish them for their past crimes”).

**Table 1:** Rationales for imprisonment in three hypothetical countries.

**Retribution condition:** In this country, the standard rationale for imprisonment is to hold prisoners accountable and punish them for their past crimes. Imprisonment is based on a belief that some kind of moral balance will be achieved by inflicting a sufficient level of misery onto the prisoner, in proportion to the perceived seriousness of the crime they are responsible for. As a punishment, prisoners spend most of the day isolated in their cells; meals are eaten in the cell and access to reading materials or TV is severely limited. Prisoners complete 8 hours of manual labor a day, as a way of "paying back" for the wrongs they have committed. Length of sentence in this country should reflect how severely you believe the person deserves to be punished.
**Public safety condition:** In this country, the standard rationale for incarceration is to keep the law-abiding public safe from people who have committed violent crimes. Incarceration is based on a belief that dangerous people must be kept away from society, because this is the most definite way of preventing these dangerous people from committing acts of violence against citizens in the community. Individuals in custody are under strict surveillance at all times, to ensure that they do not escape from the facility, and their level of risk for violence and escape are evaluated every 3 months. Their behaviors within the prison are carefully monitored, and if they are aggressive within the prison, this behavior is recorded. Length of sentence in this country should reflect how long you think the person would continue to pose a risk to society.
**Rehabilitation condition:** In this country, the standard rationale for confinement is to rehabilitate people in a way that will make them productive and law-abiding members of society. Confinement is based on a belief that criminals should undergo a rehabilitation process to change their current problematic behavior and improve their future behavior through treatment. Confinement consists of placing criminals in secured, inpatient hospitals, where they must engage in many hours of individual and group therapy. Through these therapies, these individuals can learn how to control their anger and how to manage and express their thoughts and feelings in a non-violent way. Length of sentence in this country should reflect how long you believe it takes for the person to be rehabilitated in an inpatient hospital.

*Note*: Participants in the control condition did not read any passage at this point.

Participants in the control condition did not receive any “country” vignette but instead skipped ahead to an attention check. They were told that some participants had read about a certain country and they were asked to indicate whether they had also read such information (see S1 File for attention checks).

In all four conditions, participants who answered any of the attention checks incorrectly were given the opportunity to retake the questions. If they answered any of the questions incorrectly on their second attempt, they were excluded from the analyses below (*n* = 1).

Next, all participants read a vignette, adapted with minor changes from Aspinwall et al. [22], which described a criminal case where a man was found guilty of a violent assault. The following shows an excerpt from the passage (See S1 File for the full text):

A man named J.D. (age 24 at the time) entered a restaurant at 10 PM on August 5, 2018, holding a loaded, semi-automatic gun. He demanded money from the restaurant manager, who was standing behind the counter…When the manager did not initially respond to the demand for money, J.D. forced the manager to his knees and then struck him forcefully and repeatedly in the back of the head with the gun.…J.D. was eventually arrested and confessed to assaulting the manager at the restaurant.…During the trial, J.D. was charged with assault in the 1st degree (causing serious physical harm to another by using a deadly weapon) and armed robbery (illegal taking of property in the presence of a person by violence or intimidation). In September 2019, a jury found J.D. guilty beyond a reasonable doubt of assault in the 1st degree, but he was acquitted of armed robbery as the evidence pointed to his leaving the restaurant without any money.

After reading this passage, participants again received a brief attention check, which asked them to determine whether two facts about the case were true or false (e.g., According to the passage, J.D. was found guilty of assault in the 1st degree–True or False). As with all other attention checks, participants were permitted to answer these questions a second time, if they did not answer them correctly on their first attempt. No participant failed to provide the correct answers to these questions on their second attempt.

Then, we presented participants with the baseline or pretest measure of their sentencing judgements. Specifically, participants were told that,

In the place where this crime occurred, assault in the 1st degree is characterized by causing serious physical harm to another person by using a deadly weapon. According to the law, those who commit this crime may serve a minimum sentence of 5 years and a maximum sentence of 20 years. The average sentence length for assault in the 1st degree is 8 years.…Given his assault conviction, how many years should J.D. serve?

Participants assigned to one of the experimental conditions were also instructed to consider the information they read about how their assigned country conceptualizes imprisonment. All participants then provided their pretest sentencing recommendation using a slider scale from 5 to 20 years, with one-year increments. The minimum (5 years) and maximum (20 years) sentences on this scale were based on the minimum and maximum sentences for assault in the state of Connecticut, where this research took place, see [48]. We also provided participants with an alleged average sentence length of eight years, as was used in previous studies, see [18].

Following this, all participants received a passage attributing the defendant’s violent behavior to neurobiological factors. This vignette also was adapted from Aspinwall and colleagues [22] and was described as the testimony from an independent expert. The testimony included evidence that the defendant had both genetic and neuroanatomical risk factors for violent, antisocial behavior. The original vignette from Aspinwall and colleagues [22] described how the defendant had “psychopathy”. Given the potential for lay participants to hold preconceived notions about the dangerousness and immutability of psychopathy, this was changed to “antisocial behavior” in the version below. Several lines that described the neurobiology of psychopathy also were removed. Additionally, any proper names in the passage were changed to initials, to prevent participants from making assumptions about where the crime and trial took place. Specifically, participants read the following:

The defense called Dr. R.H. to testify. Dr. H., a neurobiologist and renowned expert on the causes of antisocial behavior, testified that antisocial behavior results from genetic factors and from structural abnormalities in specific brain regions involved in emotion processing. At the genetic level, Dr. H. testified that several peer-reviewed publications reported that a polymorphism in the monoamine oxidase A (MAOA) gene, or the “warrior gene”, predicts the tendency towards antisocial behavior, particularly aggressive behavior. Dr. H. continued by saying that at the request of the defense, he genetically tested J.D. and that this test showed the MAOA polymorphism. This was evidence of J.D.’s increased risk of engaging in aggressive behavior. As further evidence, Dr. H. reported on the research that indicates this MAOA polymorphism is linked to structural abnormalities in the amygdala—a brain region involved in emotional processing and learning. Research has shown that antisocial individuals display reduced amygdala volume compared with healthy individuals. Dr. H. pointed to an MRI of J.D.’s brain, which revealed reduced amygdala volume. Dr. H. concluded that the combination of genetic and neurobiological factors ultimately interact and can lead to chronic antisocial behavior. Dr. H. noted that J.D. had been involved in the criminal justice system since he was a teenager, suggesting that the neurobiological factors described above were also detrimental throughout J.D.’s development.

After reading this testimony, all participants responded to four True/False attention check questions (e.g., An MRI of J.D.’s brain did NOT reveal reduced amygdala volume–True/False). Following the same procedure described above, participants who answered any of the questions incorrectly were permitted to retake the questions. We subsequently excluded from our analyses any participant who still answered a question incorrectly on their second attempt (*n* = 9).

Then, all participants provided their posttest sentencing recommendation for the defendant. Participants were asked, “Given [the stated goals of the criminal justice system in the country where the crime and trial took place (to punish wrongdoers; to keep the public safe; to rehabilitate criminals) and] the expert testimony about J.D.’s genetic risk for antisocial behavior and his brain’s reduced amygdala volume, what length of [sentence] would you recommend for J.D.?” Participants were also reminded of their pretest sentencing recommendations and participants assigned to the experimental conditions were reminded of the stated rationale for criminal justice that they had read about earlier, as indicated by the phrases in brackets. After reading these instructions, participants provided their final sentencing recommendations, using the same 5–20-year scale.

Following the experimental manipulation, we asked all participants to rate the extent to which they believed that their final sentencing decisions were impacted by the neuroscientific testimony. In addition, we asked all participants to rate the extent to which they agreed with each of the three rationales for incarceration described in the study. Lastly, participants completed several individual difference measures. These included questions from the aggression and drug use subscales of the Risky, impulsive, and self-destructive behavior questionnaire (RISQ) [49] a self-report measure of risky behavior across multiple domains. Engagement in risky behaviors could have led participants to be justice-involved or could have influenced how participants judged the behaviors described in the vignette, which in turn could impact their responses to the study’s primary outcome measures. Participants also responded to several demographic questions and questions about their experiences with the criminal legal system (e.g., whether the participant or a family member had ever been the victim of a crime, or convicted of a crime). The data from these measures were used in our exploratory analyses to examine the effect of any individual difference variables and the robustness of our effects.

## Results

### Sample characteristics

Ten participants were excluded for failing to correctly answer an attention check on their second attempt. An additional eight participants were excluded for failing to meet the minimum education requirement. The final sample included 784 participants (51.4% female, 47.6% male, 0.3% non-binary, 0.8% unreported, ranging in age from 18 to 83; *M* = 44.29, *SD* = 14.05). Of these participants, 27.6% were politically conservative or leaned conservative, 21.2% were moderate and 51.2% were liberal or leaned liberal. Participants did not differ on key demographics (gender, age, education level, political orientation) across the four conditions, as detailed in the S1 File.

### Effects of condition

**Fig 2:**
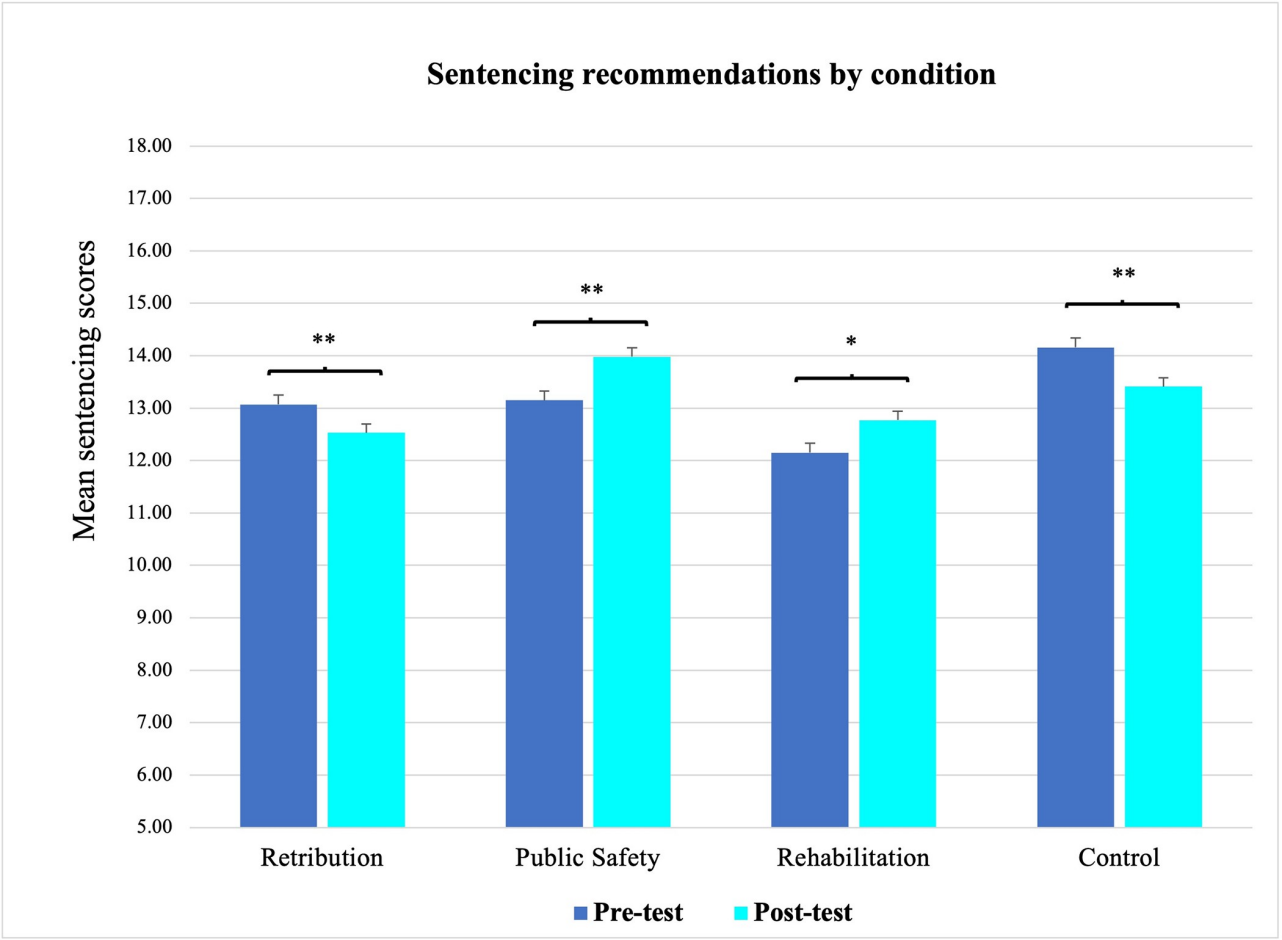
Mean sentencing estimates, broken down by condition.

Data are deposited at https://osf.io/c5vr4/. IBM SPSS Statistics Version 28 was used for statistical analyses. We examined whether participants’ sentencing estimates differed across the four conditions. A 2x4 mixed ANOVA with one within-subjects factor (timepoint: pretest vs. post-test) and one between-subjects factor (conditions) revealed no significant main effect of timepoint, *F*(1, 774) = .20, *p* = .653, but a significant main effect of condition, *F*(3, 774) = 3.89, *p* = .009, η^2^_*p*_ = .02. This main effect was qualified by a significant interaction effect *F*(3, 774) = 19.64, *p* < .001, η^2^_*p*_ = .07. Given this interaction effect, we subsequently examined sentencing estimates among participants in each condition separately (see Fig 2).

#### Retribution condition

Sentencing estimates among participants (*n* = 198) in the retribution condition decreased significantly from pretest (*M* = 13.07, *SD* = 4.39) to post-test (*M* = 12.53, *SD* = 4.59), *t*(197) = 3.95, *p* < .001, *d* = .27. These findings support our hypothesis that participants would suggest shorter sentences after they read the neuroscience testimony and after they were primed to consider retribution as the rationale for incarceration.

#### Public safety condition

Sentencing estimates among participants (*n* = 197) in the condition focused on protecting the public from dangerousness increased significantly from pretest (*M* = 13.15, *SD* = 4.52) to post-test (*M* = 13.98, *SD* = 4.80), *t*(196) = -4.09, *p* < .001, *d* = .30. These results support our hypothesis that participants primed to consider the importance of public safety would issue longer sentences after learning about the neurobiological underpinnings of a defendant’s violent behavior.

#### Rehabilitation condition

Sentencing estimates among participants (*n* = 198) in the rehabilitation condition increased significantly from pretest (*M* = 12.15, *SD* = 4.26) to post-test (*M* = 12.77, *SD* = 4.72), *t*(197) = -3.19, *p* = .002, *d* = .23. These results support our hypothesis that participants would lengthen their sentencing recommendations after reading the neuroscience testimony, when primed to consider prison to be a way of rehabilitating those convicted of crimes.

#### Control condition

Sentencing judgements among participants (*n* = 185) in the control condition decreased significantly from pretest (*M* = 14.16, *SD* = 4.54) to post-test, after they received the neuroscience testimony about the criminal J.D. (*M* = 13.41, *SD* = 4.88), *t*(184) = 4.20, *p* < .001, *d* = .31.

### Robustness analyses

For the three experimental conditions, we examined the effect of participants’ level of agreement with the stated imprisonment rationale in the condition to which they were assigned. We included this rating as a covariate in our omnibus model with one within-subjects factor (timepoint) and one between-subjects factor (conditions). The interaction effect remained significant, *F*(2, 588) = 15.00, *p* < .001, η^2^_*p*_ = .049. Additional exploratory analyses are presented in the S1 File.

## Discussion

### Summary of results

We suggest that asking whether neuroscientific explanations for criminal behavior increases or decreases the duration of sentencing is not the right question. Instead, the question should be; under what conditions do neuroscientific explanations increase or decrease the duration of sentencing? The present study found one such moderating factor: the effect of neuroscientific explanations depends on the proposed purpose of the criminal legal system. Thus, it makes sense that previous studies which did not consider this factor have found inconsistent effects regarding the impact of neuroscientific evidence on mock sentencing decisions. While some studies reported null effects of neuroscientific evidence, others found a mitigating effect, and some documented aggravating effects of such evidence. We propose that these mixed results occur because there are different effects of neuroscience explanations operating depending on the given purpose of incarceration.

In the present study, when participants imagined that the primary aim of incarceration was retribution, neuroscientific testimony about a defendant decreased sentences. This decrease likely reflected participants’ beliefs that a person with biological risk factors for antisocial behavior would be less in control of their behavior [35–37] and therefore less responsible for their actions and deserving of retribution. By contrast, when participants imagined that the primary aim of imprisonment was public safety, the neuroscientific testimony increased sentence durations, likely because biological characteristics are perceived as relatively immutable [35, 45]. That is, the defendant could be expected to continue posing a danger to society, with little likelihood of change. Similarly, when participants imagined that rehabilitation was the guiding sentencing rationale, their recommended sentences significantly increased, also reflecting participants’ beliefs that biological factors for antisocial behaviors are less mutable [35, 45].

The present study also found that participants assigned to the control condition provided significantly shorter sentences after the neuroscientific testimony than at baseline. In the absence of any specific imprisonment rationale, participants may have automatically assumed that imprisonment was a form of punishment. This would reflect the historically prevailing view of imprisonment as retributive in the United States [31]. So, when participants then received the neurobiological testimony about the defendant, they may have behaved much like participants in the retribution condition and decreased their sentence recommendations from pretest to posttest. This is a speculative interpretation however and future studies should examine how differences in one’s existing beliefs about what the current criminal legal system *is* can lead to differential effects of neuroscientific evidence.

### Related findings

Consistent with our proposal that neuroscience evidence effects are not monolithic, Allen and colleagues [40] also showed that the same neuroscience evidence could simultaneously lengthen and shorten sentences in a hypothetical case where a defendant was found guilty of a sexual assault. Though, they demonstrated these effects by using two different dependent measures (i.e., a measure of recommended prison sentence and a measure of recommended time spent in an inpatient hospital following the prison term), rather than using the same dependent measure (i.e, the sentencing scale) and experimentally manipulating the hypothesized determinant of incarceration duration as in the current study. Specifically, Allen and colleagues found that neurobiological evidence shortened the duration of prison sentencing, while it lengthened the recommended duration of involuntary hospitalization, compared to psychological evidence about the defendant’s impulse control disorder that did not mention any neurobiological causes (see [50] for similar effects). Participants who received neuroscience evidence also rated the defendant as significantly less blameworthy, deserving of punishment, acting with free will and able to stop himself, compared to participants who did not receive neuroscience evidence. These ratings help to elucidate the participants’ shorter sentence recommendations. However, to understand why Allen et al. found these effects, the researchers had to rely on correlational analyses. They did not directly manipulate the rationale used to justify the sentencing recommendations. The present study addressed the limitation of the Allen et al. study by explicitly manipulating the rationale for imprisonment.

Additionally, it is not clear from the Allen et al. study whether the longer involuntary hospitalization recommendation was obtained because neuroscience evidence caused the defendant to appear more dangerous, less treatable, or both. In fact, Allen et al. found that compared to psychological evidence, neurobiological evidence was related to rating the defendant as less dangerous. These results are puzzling as previous studies (e.g., [51]; see [52] for a review) have not found such effects. It could be that it is not that neurobiological evidence caused the defendant to appear less dangerous, but rather that psychological evidence (i.e., having an impulse control disorder) caused the defendant to appear more dangerous. Ultimately, the authors noted that “these effects [i.e., lengthened involuntary hospitalization] were not well explained by motivations…to protect society from dangerous persons.” The present study addressed this question, by separating the effect of concerns about dangerousness (i.e., the public safety condition) from concerns about the need for rehabilitation (i.e., the rehabilitation condition). Concerns about public safety and about rehabilitation both led participants to recommend longer sentences.

### Implications

The present findings have important implications for our society where science and legal philosophy continuously shift. For instance, the rehabilitation model of imprisonment has been heralded as a more humane approach within the criminal legal system [53]. Yet if more people subscribe to this approach, this might in fact lead to longer sentences for defendants who present neuroscientific evidence, as people appear to assume that defendants with biologically based problems are less able to change their behaviors. Such an increase in sentencing would be cause for concern, especially since despite public perceptions there is little scientific evidence that biologically based problems are more immutable.

Our findings also suggest that progress in neuroscience could entail an overall reduction in the duration of imprisonment under the retribution model. One possibility is that because antisocial behaviors could increasingly be explained in terms of biological mechanisms, defendants would be perceived as less accountable for their behaviors. Of course, in reality there is a larger issue that not all defendants have equal access to resources that would allow for neuroscientific evidence to be presented. Purely from a cognitive and philosophical perspective, though, the use of neuroscientific evidence could be one strategy for a slightly more humane sentencing approach under a retribution model.

To further determine what needs to be done in response to the potential consequences of neuroscience evidence presented in court, it is important to consider whether the judgments revealed in the current study are valid. In some sense, *any* changes in sentence duration recommendations upon encountering neuroscientific evidence are flawed, because all behaviors are ultimately based in neurobiology. So the time it takes to rehabilitate someone, for instance, should not necessarily depend on whether we happen to have neuroscientific evidence. That is, if one endorses that all activities of the mind are determined by the brain, then there should not be any increase or decrease in sentence duration, regardless of whether the behaviors are biologically construed.

We speculate that the reason why neuroscience evidence has any impact on sentencing, as shown in the current study, is because people implicitly or explicitly endorse mind-brain dualism, namely, believing that the mind and the brain operate somewhat independently [54]. Mind-brain dualism is entrenched in our culture, emerging early in development [55, 56] and occurring across many cultures [57, 58] and throughout history [54]. Because of this dualism, people often forget or do not realize that neurobiology (the brain) underlies all mental or physical actions (presumed to occur in “the mind”). Consequently, upon being reminded of or learning about the role of neurobiology, their assessment of others’ behaviors changes. Therefore, perhaps what is invalid are the sentencing judgments made without neuroscience evidence, as they are made based on the flawed notion of mind-brain dualism. The debate in the field has been framed in terms of how neuroscience evidence influences legal decisions, with the implicit assumption that these decisions should not be misguided by neuroscience evidence. Yet, another way of framing the problem is whether people’s judgments without neuroscience evidence might have been misled by the dualism. From this perspective, one way to counteract this problem is to remind people that all actions and behaviors have neurobiological bases whether we know what they are.

### Limitations

There are several limitations to this study. As noted above, the present study used lay participants, although judges are typically responsible for sentencing. However, the use of lay samples can provide important insight on public opinion about the legal system, which in turn can affect legal policy [40]. Future work should examine judges or other members of the criminal legal system (e.g., prosecutors), as there may be reasons that judges would show the same or a different pattern of effects as was found in the present study.

On the one hand, judges may respond similarly to lay people, when presented with specific imprisonment rationales and neuroscientific evidence about a defendant because previous studies examining the effect of neuroscientific evidence on judges’ sentencing also showed mixed results. While a study testing U.S. judges [22] showed a decrease in judges’ sentencing recommendations as a result of neuroscience evidence, in a study of German judges [50], neuroscience evidence about a defendant did not decrease the average sentence length given to the defendant. Furthermore, there was an increase in the number of judges ordering involuntary hospitalization for the defendant [50], a commitment term which the authors note, can be much longer than a prison sentence in Germany. The latter finding suggests that judges would respond in a pattern similar to that observed in the present study if they were to adopt a rehabilitation rationale for imprisonment.

On the other hand, judges may respond differently to our materials for several reasons. Judges may hold a more homogenous view of the aims of the criminal legal system than do lay people [e.g., 59, although see also 60] which likely stems from years of training and experiences. Given this, it may be more challenging for judges to flexibly adopt a different view. Additionally, judges are held to case law precedents, statutes and other rules, and are influenced by sentencing guidelines and extra-legal forces that lay juries are not [again 60–62]. Thus, it is not clear how these factors may interact with the way neuroscience evidence influences sentencing.

Another limitation is that the findings of this study might not generalize to defendants who present with different kinds of psychological disorders. For example, people hold a strong prejudice against individuals with psychopathy and may believe they are not treatable [63]. Thus neuroscience evidence may not further lengthen sentencing even under the rehabilitation or public safety models, as the sentencing judgments made without neuroscience evidence have already reached the maximum level. Alternatively, there may be cases where the observed effects could be more robust. For instance, a heinous crime, when biologically explained, might aggravate sentences to an even greater extent, when the given rationale is public safety.

The present study is also limited in that the version of the retribution principle used in the study materials does not necessarily reflect the retribution principle held by all legal scholars. Some [see 64 for a discussion] have argued that the retribution principle should be applied regardless of the defendant’s rationality (e.g., capacity to tell right from wrong, neurological abnormalities) and even the prior criminal history. Instead, they argue that retribution should be based only on the harm caused by the crime. The retribution principle used in our materials did not necessarily reflect this version of the retribution principle; that is, we did not explicitly state that the sentencing duration should be based only on the harm and not based on the defendant’s mental and neurobiological capacities. It is an open question whether laypeople would concur with the principle of punishing wrongdoers, regardless of their rationality, when explicitly presented with it as a criterion and not reduce sentencing even after the neuroscience evidence is presented. Furthermore, it is also unclear whether the reduced sentencing in the retribution condition is because the participants considered the defendant’s rationality, following the provision of the neuroscience testimony, or some other factors.

Lastly, in addition to the three rationales for imprisonment (i.e., retribution, public safety, and rehabilitation) examined in the current study, there are other penal philosophies that we did not test. For instance, we did not consider the full range of philosophies regarding deterrence. That is, deterrence may be *specific*, to prevent recidivism, or it may serve a *general* purpose as a message to other would-be offenders not to behave as the offender did [65]. In this study, all three experimental conditions involved specific deterrence, but it is yet unknown how a principle of general deterrence would interact with neuroscience evidence. This is an important principle for future research to examine.

## Conclusion

Given that neuroscience appears to be increasingly utilized in criminal legal proceedings, it is important to understand how beliefs about imprisonment affect reasoning about neuroscientific evidence and sentencing. The current study found that neuroscientific evidence does not by itself necessarily lead to mitigated or aggravated sentences, but rather that it interacts with society’s reasons for incarceration.

## Supporting information

S1 File (DOCX)
