# Supplementary material for: The effect of neuroscientific evidence on sentencing depends on how one conceives of reasons for incarceration
Source: PLoS One. 2022 Nov 2;17(11):e0276237. doi: 10.1371/journal.pone.0276237 (PMC9629607; doi:10.1371/journal.pone.0276237)
Supplement: S1 File — (DOCX) [file pone.0276237.s001.docx]

**Supporting Information**

Table of Contents

1. Verbatim study passages and corresponding comprehension checks
   1. Condition-specific rationales for incarceration and comprehension checks
   2. Criminal case description and comprehension check
   3. Expert testimony and comprehension checks
2. Verbatim sentencing scales
3. Exploratory analyses
   1. Correlations between sentencing difference scores and reaction to testimony ratings
   2. Correlations between sentencing difference scores and individual difference variables
   3. One-way ANOVA examining effect of conditions on baseline sentencing judgements
4. Balance test

**1. Verbatim study passages and corresponding comprehension checks**

**1a. Condition-specific rationales for incarceration and comprehension checks:**

| **Retribution condition:** In this country, the standard rationale for imprisonment is to hold prisoners accountable and punish them for their past crimes. Imprisonment is based on a belief that some kind of moral balance will be achieved by inflicting a sufficient level of misery onto the prisoner, in proportion to the perceived seriousness of the crime they are responsible for. As a punishment, prisoners spend most of the day isolated in their cells; meals are eaten in the cell and access to reading materials or TV is severely limited. Prisoners complete 8 hours of manual labor a day, as a way of "paying back" for the wrongs they have committed. Length of sentence in this country should reflect how severely you believe the person deserves to be punished. |
| --- |
| **Comprehension check:** At this point, we would like to check whether all the information presented to you so far is clear to you, before you are presented with further information. Based on what you read so far, please select the option below that most closely matches how you should decide the length of sentences given to people who have been found guilty of a crime in the country you read about.   - The length of sentence should be based on the time needed to hold people accountable and punish them for their past crimes. - The length of sentence should **NOT** be based on the time needed to hold people accountable and punish them for their past crimes. |
| **Rehabilitation condition:** In this country, the standard rationale for confinement is to rehabilitate people in a way that will make them productive and law-abiding members of society. Confinement is based on a belief that criminals should undergo a rehabilitation process to change their current problematic behavior and improve their future behavior through treatment. Confinement consists of placing criminals in secured, inpatient hospitals, where they must engage in many hours of individual and group therapy. Through these therapies, these individuals can learn how to control their anger and how to manage and express their thoughts and feelings in a non-violent way. Length of sentence in this country should reflect how long you believe it takes for the person to be rehabilitated in an inpatient hospital. |
| **Comprehension check:** At this point, we would like to check whether all the information presented to you so far is clear to you, before you are presented with further information. Based on what you read so far, please select the option below that most closely matches how you should decide the length of sentences given to people who have been found guilty of a crime in the country you read about.   - The length of sentence should be based on the time needed to change people in a way that will make them productive and law-abiding members of society. - The length of sentence should **NOT** be based on the time needed to change people in a way that will make them productive and law-abiding members of society. |
| **Public safety condition:**  In this country, the standard rationale for incarceration is to keep the law-abiding public safe from people who have committed violent crimes. Incarceration is based on a belief that dangerous people must be kept away from society, because this is the most definite way of preventing these dangerous people from committing acts of violence against citizens in the community. Individuals in custody are under strict surveillance at all times, to ensure that they do not escape from the facility, and their level of risk for violence and escape are evaluated every 3 months. Their behaviors within the prison are carefully monitored, and if they are aggressive within the prison, this behavior is recorded. Length of sentence in this country should reflect how long you think the person would continue to pose a risk to society. |
| **Comprehension check:** At this point, we would like to check whether all the information presented to you so far is clear to you, before you are presented with further information. Based on what you read so far, please select the option below that most closely matches how you should decide the length of sentences given to people who have been found guilty of a crime in the country you read about.   - The length of sentence should be based on the time needed to keep the public safe from people who have committed violent crimes. - The length of sentence should **NOT** be based on the time needed to keep the public safe from people who have committed violent crimes. |
| **Control condition:** Participants in this condition did not read any passage. |
| **Comprehension check:** At this point, we would like to check whether the information presented to you so far is clear to you, before you are presented with further information. Some participants read a passage about the general principles that other countries use to inform the length of sentences for those who are found guilty of crimes. Other participants did **NOT** read such a passage. Please indicate below whether you read such a passage.   - I did **NOT** read a passage about how other countries decide sentence length for people found guilty of crimes. - I did read a passage about how other countries decide sentence length for people found guilty of crimes. |

**1b. Criminal case description and comprehension check:**

| **Case description:**  A man named J.D. (age 24 at the time) entered a restaurant at 10 PM on August 5, 2018, holding a loaded, semi-automatic gun. He demanded money from the restaurant manager, who was standing behind the counter. The manager was 48 years old at the time and had no previous relation to J.D. When the manager did not initially respond to the demand for money, **J.D. forced the manager to his knees and then struck him forcefully and repeatedly in the back of the head with the gun**. J.D. later said he struck the manager because "that fat son-of-a-bitch wouldn't stop crying." J.D. ran off without taking any money.  J.D. was eventually arrested and confessed to assaulting the manager at the restaurant. The manager's blood was also found on the gun that was found in J.D.'s car. The manager sustained moderate, permanent brain damage from the forceful blows to his head. He was in the hospital, in a coma for 20 days, but has since come out of the coma and returned to his home. However, the manager continues to have difficulty remembering many words and controlling his fine motor movement (such as holding pencils or typing). J.D. bragged about his actions at the restaurant to fellow pre-trial detainees.  During the trial, J.D. was charged with assault in the 1st degree (causing serious physical harm to another by using a deadly weapon) and armed robbery (illegal taking of property in the presence of a person by violence or intimidation). In September 2019, **a jury found J.D. guilty beyond a reasonable doubt of assault in the 1st degree**, but he was acquitted of armed robbery as the evidence pointed to his leaving the restaurant without any money. |
| --- |
| **Comprehension check:**  According to the passage you just read, J.D. entered a restaurant and repeatedly struck the restaurant manager on the head with a gun.   - True - False   According to the passage, J.D. was found guilty of assault in the 1st degree.   - True - False |
|  |

Note that the case description was adapted from Aspinwall, Brown and Tabery [1], in which the vignette was presented to a sample of U.S. State trial judges. In the present study, the vignette was revised to increase the comprehensibility for lay participants, by changing the description of the crime as “aggravated battery” to “assault in the 1st degree”. Additionally, any proper names were changed to initials, so as to prevent participants from making assumptions about where the crime took place. For example, the name of the defendant was changed from “Jonathan Donahue” to the initials J.D. and the name of the restaurant, “Burger King”, where the crime took place, was also omitted. The age of the victim was changed from 25 to 48. In the original vignette, the defendant was described as “bragging about his actions at the restaurant to jail staff.” This was changed to a description of the defendant “bragging about his actions at the restaurant to fellow pre-trial detainees”. We also removed a line describing how the defendant had a “king’s crown tattooed on his back”.

**1c. Expert testimony and comprehension checks:**

| **Expert testimony**  The defense called Dr. R.H. to testify. Dr. H., a neurobiologist and renowned expert on the causes of antisocial behavior, testified that **antisocial behavior results from genetic factors and from structural abnormalities in specific brain regions involved in emotion processing**. At the genetic  level, Dr. H. testified that several peer-reviewed publications reported that **a polymorphism in the monoamine oxidase A (MAOA) gene, or the “warrior gene”, predicts the tendency towards antisocial behavior**, particularly aggressive behavior. Dr. H. continued by saying that at the request of the defense, he genetically tested J.D. and that **this test showed the MAOA polymorphism**. This was evidence of J.D.'s increased risk of engaging in aggressive behavior. As further evidence, Dr. H. reported on the research that indicates this MAOA polymorphism is linked to structural abnormalities in the amygdala - a brain region involved in emotional processing and learning. Research has shown that antisocial individuals display reduced amygdala volume compared with healthy individuals. Dr. H. pointed to an MRI of J.D.'s brain, **which revealed reduced amygdala volume**. Dr. H. concluded that **the combination of genetic and neurobiological factors ultimately**  **interact and can lead to chronic antisocial behavior**. Dr. H. noted that J.D. had been involved in the criminal justice system since he was a teenager, suggesting that the neurobiological factors described above were also detrimental throughout J.D.'s development. |
| --- |
| **Comprehension checks:** We would like to check whether the expert testimony is clear to you. On this page, please determine whether or not each sentence is consistent with what the expert stated. If any of your answers is incorrect, you will have an opportunity to see the testimony again and correct your answers.  Antisocial behavior can result from genetic factors and structural abnormalities in brain regions involved in emotion processing.   - - True   - False   A genetic test revealed that J.D. had a polymorphism in the monoamine oxidase A (MAOA) gene, or the “warrior gene”, that predicts the tendency towards antisocial (particularly aggressive) behavior.   - - True   - False   An MRI of J.D.’s brain did NOT reveal reduced amygdala volume.   - - True   - False   Genetic and neurobiological factors can interact and lead to chronic antisocial behavior.   - - True - False |

Note that the expert testimony vignette was also adapted from Aspinwall et al. [1]. The original vignette described how the defendant had “psychopathy”. Given the potential for lay participants to hold preconceived notions about psychopathy, this was changed to “antisocial behavior”. Several lines that described the neurobiology of psychopathy were also removed. Additionally, any proper names in this passage were changed to initials, to prevent participants from making assumptions about where the crime and trial took place.

**2. Verbatim sentencing scales**

| In the place where this crime occurred, assault in the 1st degree is characterized by causing serious physical harm to another person by using a deadly weapon. According to the law, those who commit this crime may serve a minimum sentence of 5 years and a maximum sentence of 20 years. The average sentence length for assault in the 1st degree is 8 years.  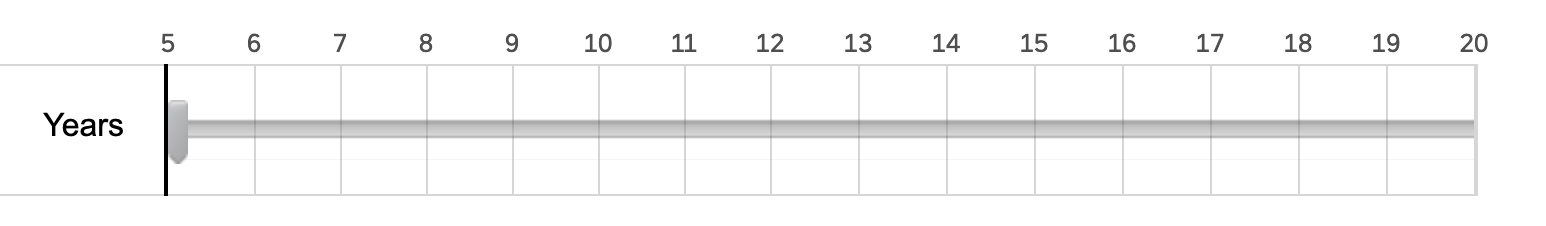 |
| --- |
| **Pretest question phrasing:** Given his assault conviction, how many years should J.D. serve? [Please keep in mind the information you have read about how this country handles criminals.]  **Posttest question phrasing:** Given the [stated goals of the criminal justice system in the country where the crime and trial took place (___________)] and the expert testimony about J.D.'s genetic risk for antisocial behavior and his brain's reduced amygdala volume, what length of [imprisonment/confinement/incarceration/prison] time would you recommend to rehabilitate J.D.?  As a reminder, your earlier sentencing recommendation was ____ years, however you should feel free to increase, decrease, or keep your final sentencing recommendation the same now that you have read the expert's testimony.  Please provide your final sentencing recommendation by using the slider below. Again, you should feel free to change, or not change your final sentencing recommendation from the original recommendation you provided. |

The sentencing scale used one-year increments with five years as the minimum possible sentence and 20 years as the maximum possible sentence. The minimum and maximum sentences are based on the minimum and maximum sentences for assault in the state of Connecticut, where this research study took place [2]. The average sentence length of eight years was used in previous studies (see [3]).

Note that participants in the control condition did *not* receive the portion of text in brackets in the Table above. In addition, the blank space in parentheses is used to indicate where participants assigned to an experimental condition received a brief reminder of the rationale for imprisonment in the country that they read about (e.g., for participants in the retribution condition, this information read as follows: “to hold prisoners accountable and punish them for their past crimes.”). The words in brackets were presented selectively to participants based on the condition they were assigned to, so as to mirror the language they had read about earlier when reading about the rationale for imprisonment in a given country. Participants in the retribution condition read the word “imprisonment,” participants in the rehabilitation condition read the word “confinement,” and participants in the public safety condition read the word “incarceration.” Participants in the control condition read the word “prison.” The unbracketed blank space indicates where participants were provided with their pretest sentencing recommendation.

**3. Exploratory analyses**

**3a. Correlations between sentencing difference scores and reaction to testimony ratings**

As converging evidence that participants’ reactions to the neuroscientific testimony ratings were driving the changes in their sentencing judgments from pretest to post-test, we examined the correlations between their self-reported reactions to the testimony and their sentencing difference scores. In the control and the retribution conditions, the greater reduction participants made in sentencing judgments from pretest to post-test, the more participants reported believing that the neuroscience decreased their final sentencing recommendations, *r*(182) = .80, *p* < .001, and *r*(196) = .76, *p* < .001, respectively. Similarly, in the public safety and the rehabilitation conditions, the more participants felt that the neuroscientific testimony increased their final sentencing recommendation, the more their scores increased from pretest to post-test, *r*(195) = .79, *p* < .001, and *r*(196) = .78, *p* < .001, respectively.

**3b. Correlations between sentencing difference scores and individual difference variables**

This section discusses the results of any significant correlations found between the sentencing difference scores (i.e., posttest scores minus pretest scores) and measures of individual differences. Correlations not discussed here were not significant. See Tables 4-7 below for all correlations. Individual difference variables were included because it is possible that demographics, real-world engagement in antisocial behavior, and/or experiences with the criminal legal system can affect how one considers neuroscience evidence and the criminal legal system. For verbatim questions used to create the individual difference variables see “Measured Variables” and “Manipulated Variables” files available at https://[osf.io/c5vr4](https://osf.io/c5vr4).

| **Table 4.** Control condition correlations with sentencing scores and individual difference variables | | | | | | | | | |  |  |  |  |  |  |  |  |  |  |  |  |  |  |  |  |
| --- | --- | --- | --- | --- | --- | --- | --- | --- | --- | --- | --- | --- | --- | --- | --- | --- | --- | --- | --- | --- | --- | --- | --- | --- | --- |
|  | | | Sentencing Difference Scores | Gender | Race | Age | Schooling | Citizen-ship Status | Political Ideology | | | Antisocial Scale Drug Abuse | Antisocial Scale Aggression | Antisocial Scale Total Score | Jury Service | Law Occupa-tion | Arrested | Self Victim Crime | Self Type of Crime | Fam Victim Crime | Fam Type of Crime | Self Convicted | Self Convict Crime Type | Fam Convicted | Fam Convict Crime Type |
| **Sentencing Difference Scores** | | Pearson Correlation | -- |  |  |  |  |  |  | | |  |  |  |  |  |  |  |  |  |  |  |  |  |  |
|  |  | N | 185 |  |  |  |  |  |  | | |  |  |  |  |  |  |  |  |  |  |  |  |  |  |
| **Gender** | | Pearson Correlation | .123 | -- |  |  |  |  |  | | |  |  |  |  |  |  |  |  |  |  |  |  |  |  |
|  |  | Sig. (2-tailed) | .097 |  |  |  |  |  |  | | |  |  |  |  |  |  |  |  |  |  |  |  |  |  |
|  |  | N | 183 | 184 |  |  |  |  |  | | |  |  |  |  |  |  |  |  |  |  |  |  |  |  |
| **Race** | | Pearson Correlation | .075 | .105 | -- |  |  |  |  | | |  |  |  |  |  |  |  |  |  |  |  |  |  |  |
|  |  | Sig. (2-tailed) | .332 | .174 |  |  |  |  |  | | |  |  |  |  |  |  |  |  |  |  |  |  |  |  |
|  |  | N | 169 | 169 | 170 |  |  |  |  | | |  |  |  |  |  |  |  |  |  |  |  |  |  |  |
| **Age** | | Pearson Correlation | .138 | -.147^*^ | .230^**^ | -- |  |  |  | | |  |  |  |  |  |  |  |  |  |  |  |  |  |  |
|  |  | Sig. (2-tailed) | .061 | .046 | .003 |  |  |  |  | | |  |  |  |  |  |  |  |  |  |  |  |  |  |  |
|  |  | N | 185 | 184 | 170 | 186 |  |  |  | | |  |  |  |  |  |  |  |  |  |  |  |  |  |  |
| **Schooling** | | Pearson Correlation | -.043 | .003 | -.003 | -.031 | -- |  |  | | |  |  |  |  |  |  |  |  |  |  |  |  |  |  |
|  |  | Sig. (2-tailed) | .564 | .966 | .971 | .674 |  |  |  | | |  |  |  |  |  |  |  |  |  |  |  |  |  |  |
|  |  | N | 185 | 184 | 170 | 186 | 186 |  |  | | |  |  |  |  |  |  |  |  |  |  |  |  |  |  |
| **Citizenship status** | | Pearson Correlation | .220^**^ | .061 | .154^*^ | -.008 | -.014 | -- |  | | |  |  |  |  |  |  |  |  |  |  |  |  |  |  |
|  |  | Sig. (2-tailed) | .003 | .411 | .046 | .910 | .854 |  |  | | |  |  |  |  |  |  |  |  |  |  |  |  |  |  |
|  |  | N | 184 | 183 | 169 | 185 | 185 | 185 |  | | |  |  |  |  |  |  |  |  |  |  |  |  |  |  |
| **Political Ideology** | | Pearson Correlation | -.121 | .037 | -.254^**^ | -.140 | -.048 | .017 | -- | | |  |  |  |  |  |  |  |  |  |  |  |  |  |  |
|  |  | Sig. (2-tailed) | .102 | .619 | <.001 | .056 | .516 | .823 |  | | |  |  |  |  |  |  |  |  |  |  |  |  |  |  |
|  |  | N | 185 | 184 | 170 | 186 | 186 | 185 | 186 | | |  |  |  |  |  |  |  |  |  |  |  |  |  |  |
| **Antisocial Scale - DrugAbuse** | | Pearson Correlation | .036 | .199^**^ | .093 | .022 | -.162^*^ | .048 | .171^*^ | | | -- |  |  |  |  |  |  |  |  |  |  |  |  |  |
|  |  | Sig. (2-tailed) | .625 | .007 | .229 | .767 | .027 | .515 | .019 | | |  |  |  |  |  |  |  |  |  |  |  |  |  |  |
|  |  | N | 185 | 184 | 170 | 186 | 186 | 185 | 186 | | | 186 |  |  |  |  |  |  |  |  |  |  |  |  |  |
| **Antisocial Scale - Aggression** | | Pearson Correlation | .119 | .447^**^ | -.023 | -.099 | -.156^*^ | .062 | .081 | | | .362^**^ | -- |  |  |  |  |  |  |  |  |  |  |  |  |
|  |  | Sig. (2-tailed) | .108 | <.001 | .761 | .178 | .034 | .405 | .270 | | | <.001 |  |  |  |  |  |  |  |  |  |  |  |  |  |
|  |  | N | 185 | 184 | 170 | 186 | 186 | 185 | 186 | | | 186 | 186 |  |  |  |  |  |  |  |  |  |  |  |  |
| **Antisocial Scale – Total Score** | | Pearson Correlation | .067 | .304^**^ | .072 | -.011 | -.185^*^ | .060 | .170^*^ | | | .960^**^ | .609^**^ | -- |  |  |  |  |  |  |  |  |  |  |  |
|  |  | Sig. (2-tailed) | .368 | <.001 | .351 | .879 | .012 | .421 | .020 | | | <.001 | <.001 |  |  |  |  |  |  |  |  |  |  |  |  |
|  |  | N | 185 | 184 | 170 | 186 | 186 | 185 | 186 | | | 186 | 186 | 186 |  |  |  |  |  |  |  |  |  |  |  |
| **Jury Service** | | Pearson Correlation | .027 | .025 | -.021 | .207^**^ | .117 | .036 | .032 | | | .000 | -.064 | -.020 | -- |  |  |  |  |  |  |  |  |  |  |
|  |  | Sig. (2-tailed) | .718 | .733 | .782 | .005 | .113 | .624 | .665 | | | .995 | .385 | .790 |  |  |  |  |  |  |  |  |  |  |  |
|  |  | N | 185 | 184 | 170 | 186 | 186 | 185 | 186 | | | 186 | 186 | 186 | 186 |  |  |  |  |  |  |  |  |  |  |
| **Law Occupation** | | Pearson Correlation | .046 | .027 | .078 | -.032 | -.007 | .011 | -.090 | | | -.007 | .045 | .007 | .204^**^ | -- |  |  |  |  |  |  |  |  |  |
|  |  | Sig. (2-tailed) | .534 | .713 | .313 | .665 | .926 | .882 | .223 | | | .923 | .547 | .921 | .005 |  |  |  |  |  |  |  |  |  |  |
|  |  | N | 185 | 183 | 169 | 185 | 185 | 184 | 185 | | | 185 | 185 | 185 | 185 | 185 |  |  |  |  |  |  |  |  |  |
| **Arrested** | | Pearson Correlation | .040 | .073 | .054 | .038 | -.152^*^ | .028 | .074 | | | .565^**^ | .273^**^ | .563^**^ | -.031 | -.057 | -- |  |  |  |  |  |  |  |  |
|  |  | Sig. (2-tailed) | .589 | .326 | .487 | .604 | .039 | .701 | .313 | | | <.001 | <.001 | <.001 | .673 | .438 |  |  |  |  |  |  |  |  |  |
|  |  | N | 185 | 184 | 170 | 186 | 186 | 185 | 186 | | | 186 | 186 | 186 | 186 | 185 | 186 |  |  |  |  |  |  |  |  |
| **Self Victim of Crime** | | Pearson Correlation | .104 | .068 | .082 | .191^**^ | -.143 | .079 | .020 | | | .245^**^ | .220^**^ | .275^**^ | .116 | .064 | .200^**^ | -- |  |  |  |  |  |  |  |
|  |  | Sig. (2-tailed) | .157 | .361 | .285 | .009 | .052 | .285 | .783 | | | <.001 | .003 | <.001 | .114 | .386 | .006 |  |  |  |  |  |  |  |  |
|  |  | N | 185 | 184 | 170 | 186 | 186 | 185 | 186 | | | 186 | 186 | 186 | 186 | 185 | 186 | 186 |  |  |  |  |  |  |  |
| **Self Type of Crime** | | Pearson Correlation | .146 | -.126 | -.212^*^ | -.093 | -.193 | .^b^ | -.056 | | | -.157 | .041 | -.119 | -.111 | -.004 | -.028 | .^b^ | -- |  |  |  |  |  |  |
|  |  | Sig. (2-tailed) | .150 | .216 | .046 | .358 | .056 | . | .581 | | | .120 | .686 | .239 | .273 | .971 | .780 | . |  |  |  |  |  |  |  |
|  |  | N | 99 | 98 | 89 | 99 | 99 | 99 | 99 | | | 99 | 99 | 99 | 99 | 99 | 99 | 99 | 99 |  |  |  |  |  |  |
| **Family Victim of Crime** | | Pearson Correlation | .078 | .121 | .024 | .057 | .065 | .082 | -.005 | | | .161^*^ | .296^**^ | .226^**^ | .176^*^ | .133 | .120 | .437^**^ | .062 | -- |  |  |  |  |  |
|  |  | Sig. (2-tailed) | .291 | .103 | .759 | .443 | .379 | .269 | .943 | | | .028 | <.001 | .002 | .016 | .072 | .104 | <.001 | .544 |  |  |  |  |  |  |
|  |  | N | 185 | 184 | 170 | 186 | 186 | 185 | 186 | | | 186 | 186 | 186 | 186 | 185 | 186 | 186 | 99 | 186 |  |  |  |  |  |
| **Fam Type of Crime** | | Pearson Correlation | .073 | .029 | -.292^**^ | .046 | -.133 | .^b^ | .005 | | | .160 | .206^*^ | .199^*^ | .021 | .030 | .250^*^ | .042 | .342^**^ | .^b^ | -- |  |  |  |  |
|  |  | Sig. (2-tailed) | .465 | .774 | .004 | .644 | .180 | . | .962 | | | .107 | .037 | .044 | .835 | .767 | .011 | .671 | .003 | . |  |  |  |  |  |
|  |  | N | 103 | 102 | 94 | 103 | 103 | 102 | 103 | | | 103 | 103 | 103 | 103 | 103 | 103 | 103 | 75 | 103 | 103 |  |  |  |  |
| **Self Convicted** | | Pearson Correlation | -.033 | .094 | .030 | -.031 | -.230^**^ | .016 | .047 | | | .313^**^ | .216^**^ | .332^**^ | .027 | -.032 | .472^**^ | .146^*^ | .049 | -.023 | .131 | -- |  |  |  |
|  |  | Sig. (2-tailed) | .658 | .203 | .702 | .670 | .002 | .832 | .520 | | | <.001 | .003 | <.001 | .713 | .669 | <.001 | .047 | .627 | .756 | .187 |  |  |  |  |
|  |  | N | 185 | 184 | 170 | 186 | 186 | 185 | 186 | | | 186 | 186 | 186 | 186 | 185 | 186 | 186 | 99 | 186 | 103 | 186 |  |  |  |
| **Self Convict Crime Type** | | Pearson Correlation | .^b^ | .^b^ | .^b^ | .^b^ | .^b^ | .^b^ | .^b^ | | | .^b^ | .^b^ | .^b^ | .^b^ | .^b^ | .^b^ | .^b^ | .^b^ | .^b^ | .^b^ | .^b^ | .^b^ |  |  |
|  |  | Sig. (2-tailed) | . | . | . | . | . | . | . | | | . | . | . | . | . | . | . | . | . | . | . |  |  |  |
|  |  | N | 8 | 8 | 7 | 8 | 8 | 8 | 8 | | | 8 | 8 | 8 | 8 | 8 | 8 | 8 | 7 | 8 | 4 | 8 | 8 |  |  |
| **Fam Convicted** | | Pearson Correlation | .103 | -.062 | -.118 | .001 | -.170^*^ | .039 | .108 | | | .170^*^ | .234^**^ | .216^**^ | -.031 | -.078 | .111 | .124 | .003 | .259^**^ | .184 | .212^**^ | .^b^ | -- |  |
|  |  | Sig. (2-tailed) | .162 | .405 | .127 | .988 | .020 | .601 | .144 | | | .020 | .001 | .003 | .671 | .291 | .132 | .093 | .973 | <.001 | .063 | .004 | . |  |  |
|  |  | N | 185 | 184 | 170 | 186 | 186 | 185 | 186 | | | 186 | 186 | 186 | 186 | 185 | 186 | 186 | 99 | 186 | 103 | 186 | 8 | 186 |  |
| **Fam Convict Crime Type** | | Pearson Correlation | .352^*^ | .370^*^ | -.008 | .099 | -.046 | .^b^ | -.067 | | | .183 | .412^**^ | .286 | -.284 | .^b^ | .112 | .217 | -.190 | .308 | .240 | -.063 | .^b^ | .^b^ | -- |
|  |  | Sig. (2-tailed) | .026 | .019 | .963 | .543 | .779 | . | .681 | | | .257 | .008 | .074 | .076 | . | .491 | .178 | .354 | .053 | .185 | .697 | . | . |  |
|  |  | N | 40 | 40 | 34 | 40 | 40 | 40 | 40 | | | 40 | 40 | 40 | 40 | 40 | 40 | 40 | 26 | 40 | 32 | 40 | 5 | 40 | 40 |
| ** Correlation is significant at the .01 level (2-tailed)  * Correlation is significant at the .05 level (2-tailed)  b Correlation not reported because at least one of the variables is constant | | | | | | | | | |  |  |  |  |  |  |  |  |  |  |  |  |  |  |  |  |
|  |  | | | | | | | | |  |  |  |  |  |  |  |  |  |  |  |  |  |  |  |  |

**Table 5.** Retribution condition correlations with sentencing scores and individual difference variables

| **Correlations** | | | | | | | | | | | | | | | | | | | | | | |
| --- | --- | --- | --- | --- | --- | --- | --- | --- | --- | --- | --- | --- | --- | --- | --- | --- | --- | --- | --- | --- | --- | --- |
|  | | Sentencing Difference Scores | Gender | Race | Age | Schooling | Citizen | Political Ideology | Antisocial Scale Drug Abuse | Antisocial Scale Aggression | Antisocial Scale Total Score | Jury Service | Law Occupation | Arrested | Self Victim Crime | Self Type of Crime | Fam Victim Crime | Fam Type of Crime | Self Convicted | Self Convict Crime Type | Fam Convicted | Fam Convict Crime Type |
| Sentencing Difference Scores | Pearson Correlation | -- |  |  |  |  |  |  |  |  |  |  |  |  |  |  |  |  |  |  |  |  |
|  | N | 198 |  |  |  |  |  |  |  |  |  |  |  |  |  |  |  |  |  |  |  |  |
| Gender | Pearson Correlation | .121 | -- |  |  |  |  |  |  |  |  |  |  |  |  |  |  |  |  |  |  |  |
|  | Sig. (2-tailed) | .090 |  |  |  |  |  |  |  |  |  |  |  |  |  |  |  |  |  |  |  |  |
|  | N | 197 | 199 |  |  |  |  |  |  |  |  |  |  |  |  |  |  |  |  |  |  |  |
| Race | Pearson Correlation | -.057 | -.075 | -- |  |  |  |  |  |  |  |  |  |  |  |  |  |  |  |  |  |  |
|  | Sig. (2-tailed) | .443 | .307 |  |  |  |  |  |  |  |  |  |  |  |  |  |  |  |  |  |  |  |
|  | N | 186 | 188 | 188 |  |  |  |  |  |  |  |  |  |  |  |  |  |  |  |  |  |  |
| Age | Pearson Correlation | -.025 | -.094 | .237^**^ | -- |  |  |  |  |  |  |  |  |  |  |  |  |  |  |  |  |  |
|  | Sig. (2-tailed) | .725 | .187 | .001 |  |  |  |  |  |  |  |  |  |  |  |  |  |  |  |  |  |  |
|  | N | 198 | 199 | 188 | 200 |  |  |  |  |  |  |  |  |  |  |  |  |  |  |  |  |  |
| Schooling | Pearson Correlation | .123 | .049 | .061 | .038 | -- |  |  |  |  |  |  |  |  |  |  |  |  |  |  |  |  |
|  | Sig. (2-tailed) | .083 | .496 | .406 | .598 |  |  |  |  |  |  |  |  |  |  |  |  |  |  |  |  |  |
|  | N | 198 | 199 | 188 | 200 | 200 |  |  |  |  |  |  |  |  |  |  |  |  |  |  |  |  |
| Citizen | Pearson Correlation | .^a^ | .^a^ | .^a^ | .^a^ | .^a^ | .^a^ |  |  |  |  |  |  |  |  |  |  |  |  |  |  |  |
|  | Sig. (2-tailed) | . | . | . | . | . |  |  |  |  |  |  |  |  |  |  |  |  |  |  |  |  |
|  | N | 197 | 198 | 187 | 199 | 199 | 199 |  |  |  |  |  |  |  |  |  |  |  |  |  |  |  |
| Political Ideology | Pearson Correlation | -.124 | -.014 | -.094 | -.126 | -.029 | .^a^ | -- |  |  |  |  |  |  |  |  |  |  |  |  |  |  |
|  | Sig. (2-tailed) | .082 | .849 | .201 | .075 | .684 | . |  |  |  |  |  |  |  |  |  |  |  |  |  |  |  |
|  | N | 198 | 199 | 188 | 200 | 200 | 199 | 200 |  |  |  |  |  |  |  |  |  |  |  |  |  |  |
| Antisocial Scale – Drug Abuse | Pearson Correlation | -.100 | .107 | .051 | -.012 | -.270^**^ | .^a^ | .099 | -- |  |  |  |  |  |  |  |  |  |  |  |  |  |
|  | Sig. (2-tailed) | .162 | .134 | .491 | .867 | <.001 | . | .161 |  |  |  |  |  |  |  |  |  |  |  |  |  |  |
|  | N | 198 | 199 | 188 | 200 | 200 | 199 | 200 | 200 |  |  |  |  |  |  |  |  |  |  |  |  |  |
| Antisocial Scale –Aggression | Pearson Correlation | -.060 | .312^**^ | -.004 | -.019 | -.189^**^ | .^a^ | -.013 | .573^**^ | -- |  |  |  |  |  |  |  |  |  |  |  |  |
|  | Sig. (2-tailed) | .401 | <.001 | .957 | .785 | .007 | . | .855 | <.001 |  |  |  |  |  |  |  |  |  |  |  |  |  |
|  | N | 198 | 199 | 188 | 200 | 200 | 199 | 200 | 200 | 200 |  |  |  |  |  |  |  |  |  |  |  |  |
| Antisocial Scale – Total Score | Pearson Correlation | -.098 | .173^*^ | .040 | -.015 | -.273^**^ | .^a^ | .078 | .974^**^ | .744^**^ | -- |  |  |  |  |  |  |  |  |  |  |  |
|  | Sig. (2-tailed) | .170 | .014 | .584 | .832 | <.001 | . | .275 | <.001 | <.001 |  |  |  |  |  |  |  |  |  |  |  |  |
|  | N | 198 | 199 | 188 | 200 | 200 | 199 | 200 | 200 | 200 | 200 |  |  |  |  |  |  |  |  |  |  |  |
| Jury Service | Pearson Correlation | .129 | .015 | -.013 | .298^**^ | -.026 | .^a^ | .140^*^ | -.013 | .098 | .017 | -- |  |  |  |  |  |  |  |  |  |  |
|  | Sig. (2-tailed) | .069 | .834 | .863 | <.001 | .714 | . | .048 | .857 | .168 | .815 |  |  |  |  |  |  |  |  |  |  |  |
|  | N | 198 | 199 | 188 | 200 | 200 | 199 | 200 | 200 | 200 | 200 | 200 |  |  |  |  |  |  |  |  |  |  |
| Law Occupation | Pearson Correlation | .113 | -.028 | .076 | .018 | .028 | .^a^ | -.010 | .071 | .120 | .091 | .124 | -- |  |  |  |  |  |  |  |  |  |
|  | Sig. (2-tailed) | .113 | .694 | .298 | .798 | .692 | . | .893 | .320 | .092 | .201 | .080 |  |  |  |  |  |  |  |  |  |  |
|  | N | 198 | 199 | 188 | 200 | 200 | 199 | 200 | 200 | 200 | 200 | 200 | 200 |  |  |  |  |  |  |  |  |  |
| Arrested | Pearson Correlation | -.109 | .025 | .075 | .103 | -.146^*^ | .^a^ | -.079 | .480^**^ | .433^**^ | .511^**^ | -.017 | -.061 | -- |  |  |  |  |  |  |  |  |
|  | Sig. (2-tailed) | .127 | .729 | .307 | .146 | .039 | . | .266 | <.001 | <.001 | <.001 | .809 | .395 |  |  |  |  |  |  |  |  |  |
|  | N | 198 | 199 | 188 | 200 | 200 | 199 | 200 | 200 | 200 | 200 | 200 | 200 | 200 |  |  |  |  |  |  |  |  |
| Self Victim Crime | Pearson Correlation | .074 | -.015 | .032 | .275^**^ | -.004 | .^a^ | -.063 | .192^**^ | .151^*^ | .198^**^ | .080 | .035 | .113 | -- |  |  |  |  |  |  |  |
|  | Sig. (2-tailed) | .300 | .828 | .662 | <.001 | .961 | . | .375 | .006 | .033 | .005 | .259 | .620 | .110 |  |  |  |  |  |  |  |  |
|  | N | 198 | 199 | 188 | 200 | 200 | 199 | 200 | 200 | 200 | 200 | 200 | 200 | 200 | 200 |  |  |  |  |  |  |  |
| Self Typeof Crime | Pearson Correlation | -.145 | -.056 | .013 | -.187 | -.180 | .^a^ | -.026 | .245^*^ | .145 | .245^*^ | -.097 | -.130 | .084 | .^a^ | -- |  |  |  |  |  |  |
|  | Sig. (2-tailed) | .157 | .584 | .899 | .065 | .076 | . | .803 | .015 | .154 | .015 | .343 | .204 | .411 | . |  |  |  |  |  |  |  |
|  | N | 97 | 98 | 92 | 98 | 98 | 97 | 98 | 98 | 98 | 98 | 98 | 98 | 98 | 98 | 98 |  |  |  |  |  |  |
| Fam Victim Crime | Pearson Correlation | .013 | -.086 | -.085 | -.024 | -.120 | .^a^ | .036 | .122 | .092 | .125 | -.005 | -.027 | .026 | .370^**^ | -.178 | -- |  |  |  |  |  |
|  | Sig. (2-tailed) | .859 | .227 | .248 | .733 | .091 | . | .611 | .085 | .194 | .078 | .944 | .702 | .710 | <.001 | .079 |  |  |  |  |  |  |
|  | N | 198 | 199 | 188 | 200 | 200 | 199 | 200 | 200 | 200 | 200 | 200 | 200 | 200 | 200 | 98 | 200 |  |  |  |  |  |
| Fam Type of Crime | Pearson Correlation | .023 | -.145 | -.006 | -.079 | -.046 | .^a^ | -.180 | .054 | .058 | .060 | -.172 | -.114 | .003 | .038 | .515^**^ | .^a^ | -- |  |  |  |  |
|  | Sig. (2-tailed) | .823 | .155 | .954 | .440 | .657 | . | .078 | .603 | .571 | .561 | .093 | .266 | .980 | .715 | <.001 | . |  |  |  |  |  |
|  | N | 96 | 97 | 91 | 97 | 97 | 97 | 97 | 97 | 97 | 97 | 97 | 97 | 97 | 97 | 66 | 97 | 97 |  |  |  |  |
| Self Convicted | Pearson Correlation | -.111 | .118 | .016 | .070 | -.208^**^ | .^a^ | -.020 | .481^**^ | .406^**^ | .504^**^ | -.049 | -.047 | .724^**^ | .080 | .038 | .119 | .121 | -- |  |  |  |
|  | Sig. (2-tailed) | .118 | .096 | .832 | .325 | .003 | . | .775 | <.001 | <.001 | <.001 | .493 | .507 | <.001 | .262 | .713 | .092 | .239 |  |  |  |  |
|  | N | 198 | 199 | 188 | 200 | 200 | 199 | 200 | 200 | 200 | 200 | 200 | 200 | 200 | 200 | 98 | 200 | 97 | 200 |  |  |  |
| Self Convict Crime Type | Pearson Correlation | .217 | .255 | -.409 | -.113 | -.202 | .^a^ | -.325 | .065 | .160 | .086 | -.182 | .^a^ | .098 | -.098 | .408 | -.153 | .289 | .^a^ | -- |  |  |
|  | Sig. (2-tailed) | .420 | .341 | .165 | .676 | .453 | . | .219 | .810 | .555 | .751 | .501 | . | .719 | .719 | .242 | .572 | .389 | . |  |  |  |
|  | N | 16 | 16 | 13 | 16 | 16 | 16 | 16 | 16 | 16 | 16 | 16 | 16 | 16 | 16 | 10 | 16 | 11 | 16 | 16 |  |  |
| Fam Convicted | Pearson Correlation | -.232^**^ | -.145^*^ | -.051 | -.035 | -.188^**^ | .^a^ | .025 | .091 | .119 | .107 | .041 | -.076 | .092 | .074 | .246^*^ | .233^**^ | .175 | .192^**^ | -.333 | -- |  |
|  | Sig. (2-tailed) | <.001 | .041 | .483 | .622 | .008 | . | .721 | .202 | .094 | .132 | .569 | .283 | .193 | .298 | .014 | <.001 | .086 | .007 | .207 |  |  |
|  | N | 198 | 199 | 188 | 200 | 200 | 199 | 200 | 200 | 200 | 200 | 200 | 200 | 200 | 200 | 98 | 200 | 97 | 200 | 16 | 200 |  |
| Fam Convict Crime Type | Pearson Correlation | .038 | -.085 | -.005 | -.271 | .092 | .^a^ | -.094 | -.049 | .074 | -.024 | -.198 | .^a^ | -.187 | -.094 | -.091 | -.098 | .138 | -.187 | .^a^ | .^a^ | -- |
|  | Sig. (2-tailed) | .825 | .621 | .977 | .105 | .588 | . | .580 | .775 | .663 | .888 | .240 | . | .267 | .578 | .694 | .562 | .492 | .267 | . | . |  |
|  | N | 36 | 36 | 32 | 37 | 37 | 37 | 37 | 37 | 37 | 37 | 37 | 37 | 37 | 37 | 21 | 37 | 27 | 37 | 7 | 37 | 37 |
| **. Correlation is significant at the 0.01 level (2-tailed). | | | | | | | | | | | | | | | | | | | | | | |
| *. Correlation is significant at the 0.05 level (2-tailed). | | | | | | | | | | | | | | | | | | | | | | |
| a. Correlation not reported because at least one of the variables is constant. | | | | | | | | | | | | | | | | | | | | | | |

**Table 6.** Public safety condition correlations with sentencing scores and individual difference variables

| **Correlations** | | | | | | | | | | | | | | | | | | | | | | | |
| --- | --- | --- | --- | --- | --- | --- | --- | --- | --- | --- | --- | --- | --- | --- | --- | --- | --- | --- | --- | --- | --- | --- | --- |
|  | | Sentencing Difference Scores | Gender | Race | Age | Schooling | Citizen | Political Ideology | Antisocial Scale Drug Abuse | Antisocial Scale Aggression | Antisocial Scale Total Score | Jury Service | Law Occupation | Arrested | | Self Victim Crime | Self Type of Crime | Fam Victim Crime | Fam Type of Crime | Self Convicted | Self Convict Crime Type | Fam Convicted | Fam Convict Crime Type |
| Sentencing Difference Scores | Pearson Correlation | -- |  |  |  |  |  |  |  |  |  |  |  | |  |  |  |  |  |  |  |  |  |
|  | N | 197 |  |  |  |  |  |  |  |  |  |  |  | |  |  |  |  |  |  |  |  |  |
| Gender | Pearson Correlation | -.200^**^ | -- |  |  |  |  |  |  |  |  |  |  | |  |  |  |  |  |  |  |  |  |
|  | Sig. (2-tailed) | .005 |  |  |  |  |  |  |  |  |  |  |  | |  |  |  |  |  |  |  |  |  |
|  | N | 194 | 196 |  |  |  |  |  |  |  |  |  |  | |  |  |  |  |  |  |  |  |  |
| Race | Pearson Correlation | -.101 | -.029 | -- |  |  |  |  |  |  |  |  |  | |  |  |  |  |  |  |  |  |  |
|  | Sig. (2-tailed) | .165 | .695 |  |  |  |  |  |  |  |  |  |  | |  |  |  |  |  |  |  |  |  |
|  | N | 190 | 190 | 192 |  |  |  |  |  |  |  |  |  | |  |  |  |  |  |  |  |  |  |
| Age | Pearson Correlation | .153^*^ | -.066 | .101 | -- |  |  |  |  |  |  |  |  | |  |  |  |  |  |  |  |  |  |
|  | Sig. (2-tailed) | .032 | .361 | .161 |  |  |  |  |  |  |  |  |  | |  |  |  |  |  |  |  |  |  |
|  | N | 197 | 196 | 192 | 199 |  |  |  |  |  |  |  |  | |  |  |  |  |  |  |  |  |  |
| Schooling | Pearson Correlation | .198^**^ | -.069 | .102 | .101 | -- |  |  |  |  |  |  |  | |  |  |  |  |  |  |  |  |  |
|  | Sig. (2-tailed) | .005 | .335 | .160 | .157 |  |  |  |  |  |  |  |  | |  |  |  |  |  |  |  |  |  |
|  | N | 197 | 196 | 192 | 199 | 199 |  |  |  |  |  |  |  | |  |  |  |  |  |  |  |  |  |
| Citizen | Pearson Correlation | -.067 | .028 | .095 | .036 | .018 | -- |  |  |  |  |  |  | |  |  |  |  |  |  |  |  |  |
|  | Sig. (2-tailed) | .354 | .695 | .190 | .613 | .802 |  |  |  |  |  |  |  | |  |  |  |  |  |  |  |  |  |
|  | N | 196 | 195 | 191 | 198 | 198 | 198 |  |  |  |  |  |  | |  |  |  |  |  |  |  |  |  |
| Political Ideology | Pearson Correlation | -.072 | -.084 | -.127 | -.127 | -.058 | .045 | -- |  |  |  |  |  | |  |  |  |  |  |  |  |  |  |
|  | Sig. (2-tailed) | .315 | .241 | .079 | .073 | .415 | .532 |  |  |  |  |  |  | |  |  |  |  |  |  |  |  |  |
|  | N | 197 | 196 | 192 | 199 | 199 | 198 | 199 |  |  |  |  |  | |  |  |  |  |  |  |  |  |  |
| Antisocial Scale – Drug Abuse | Pearson Correlation | -.023 | .079 | .113 | -.052 | -.164^*^ | .091 | .111 | -- |  |  |  |  | |  |  |  |  |  |  |  |  |  |
|  | Sig. (2-tailed) | .751 | .273 | .118 | .470 | .020 | .201 | .117 |  |  |  |  |  | |  |  |  |  |  |  |  |  |  |
|  | N | 197 | 196 | 192 | 199 | 199 | 198 | 199 | 199 |  |  |  |  | |  |  |  |  |  |  |  |  |  |
| Antisocial Scale – Aggression | Pearson Correlation | -.061 | .329^**^ | -.015 | -.108 | -.154^*^ | .023 | .081 | .232^**^ | -- |  |  |  | |  |  |  |  |  |  |  |  |  |
|  | Sig. (2-tailed) | .394 | <.001 | .836 | .131 | .030 | .753 | .256 | <.001 |  |  |  |  | |  |  |  |  |  |  |  |  |  |
|  | N | 197 | 196 | 192 | 199 | 199 | 198 | 199 | 199 | 199 |  |  |  | |  |  |  |  |  |  |  |  |  |
| Antisocial Scale – Total Score | Pearson Correlation | -.039 | .170^*^ | .097 | -.079 | -.192^**^ | .088 | .123 | .954^**^ | .513^**^ | -- |  |  | |  |  |  |  |  |  |  |  |  |
|  | Sig. (2-tailed) | .587 | .017 | .182 | .270 | .007 | .220 | .083 | <.001 | <.001 |  |  |  | |  |  |  |  |  |  |  |  |  |
|  | N | 197 | 196 | 192 | 199 | 199 | 198 | 199 | 199 | 199 | 199 |  |  | |  |  |  |  |  |  |  |  |  |
| Jury Service | Pearson Correlation | .093 | -.036 | -.014 | .364^**^ | .009 | .091 | .087 | -.013 | -.122 | -.049 | -- |  | |  |  |  |  |  |  |  |  |  |
|  | Sig. (2-tailed) | .194 | .618 | .852 | <.001 | .895 | .202 | .224 | .851 | .086 | .488 |  |  | |  |  |  |  |  |  |  |  |  |
|  | N | 197 | 196 | 192 | 199 | 199 | 198 | 199 | 199 | 199 | 199 | 199 |  | |  |  |  |  |  |  |  |  |  |
| Law Occupation | Pearson Correlation | .112 | -.180^*^ | .051 | .057 | .184^**^ | .037 | -.084 | -.018 | -.019 | -.022 | .139 | -- | |  |  |  |  |  |  |  |  |  |
|  | Sig. (2-tailed) | .119 | .012 | .487 | .429 | .010 | .603 | .239 | .798 | .790 | .758 | .051 |  | |  |  |  |  |  |  |  |  |  |
|  | N | 196 | 195 | 191 | 198 | 198 | 197 | 198 | 198 | 198 | 198 | 198 | 198 | |  |  |  |  |  |  |  |  |  |
| Arrested | Pearson Correlation | -.066 | .123 | .073 | .049 | -.133 | .055 | .005 | .445^**^ | .186^**^ | .450^**^ | .036 | .070 | | -- |  |  |  |  |  |  |  |  |
|  | Sig. (2-tailed) | .357 | .087 | .316 | .491 | .061 | .438 | .944 | <.001 | .008 | <.001 | .616 | .325 | |  |  |  |  |  |  |  |  |  |
|  | N | 197 | 196 | 192 | 199 | 199 | 198 | 199 | 199 | 199 | 199 | 199 | 198 | | 199 |  |  |  |  |  |  |  |  |
| Self Victim Crime | Pearson Correlation | -.085 | -.057 | .147^*^ | .068 | .016 | -.005 | .020 | .128 | .177^*^ | .168^*^ | .137 | .197^**^ | | .201^**^ | -- |  |  |  |  |  |  |  |
|  | Sig. (2-tailed) | .235 | .424 | .041 | .339 | .818 | .940 | .781 | .071 | .012 | .018 | .054 | .005 | | .004 |  |  |  |  |  |  |  |  |
|  | N | 197 | 196 | 192 | 199 | 199 | 198 | 199 | 199 | 199 | 199 | 199 | 198 | | 199 | 199 |  |  |  |  |  |  |  |
| Self Type of Crime | Pearson Correlation | -.072 | -.013 | -.004 | -.073 | -.131 | -.045 | .324^**^ | .201 | .125 | .220 | .009 | .182 | | -.011 | .^c^ | -- |  |  |  |  |  |  |
|  | Sig. (2-tailed) | .537 | .909 | .975 | .529 | .259 | .700 | .004 | .082 | .283 | .056 | .937 | .115 | | .924 | . |  |  |  |  |  |  |  |
|  | N | 75 | 75 | 73 | 76 | 76 | 76 | 76 | 76 | 76 | 76 | 76 | 76 | | 76 | 76 | 76 |  |  |  |  |  |  |
| Fam Victim Crime | Pearson Correlation | -.013 | .028 | .053 | -.024 | -.097 | .010 | .010 | .095 | .231^**^ | .155^*^ | .036 | .080 | | .166^*^ | .409^**^ | -.009 | -- |  |  |  |  |  |
|  | Sig. (2-tailed) | .852 | .699 | .464 | .741 | .171 | .894 | .892 | .184 | .001 | .029 | .618 | .265 | | .019 | <.001 | .937 |  |  |  |  |  |  |
|  | N | 197 | 196 | 192 | 199 | 199 | 198 | 199 | 199 | 199 | 199 | 199 | 198 | | 199 | 199 | 76 | 199 |  |  |  |  |  |
| Fam Type of Crime | Pearson Correlation | .104 | .085 | -.143 | .010 | -.207 | -.009 | -.020 | .070 | .228^*^ | .131 | -.180 | .108 | | .026 | -.071 | .611^**^ | .^c^ | -- |  |  |  |  |
|  | Sig. (2-tailed) | .351 | .446 | .199 | .927 | .057 | .934 | .858 | .524 | .036 | .233 | .098 | .324 | | .812 | .518 | <.001 | . |  |  |  |  |  |
|  | N | 83 | 83 | 82 | 85 | 85 | 85 | 85 | 85 | 85 | 85 | 85 | 85 | | 85 | 85 | 52 | 85 | 85 |  |  |  |  |
| Self Convicted | Pearson Correlation | .047 | .005 | .105 | .059 | -.078 | .037 | -.011 | .274^**^ | .066 | .262^**^ | -.076 | .052 | | .595^**^ | .056 | -.093 | .080 | .108 | -- |  |  |  |
|  | Sig. (2-tailed) | .514 | .948 | .148 | .408 | .271 | .604 | .879 | <.001 | .356 | <.001 | .287 | .466 | | <.001 | .433 | .426 | .259 | .324 |  |  |  |  |
|  | N | 197 | 196 | 192 | 199 | 199 | 198 | 199 | 199 | 199 | 199 | 199 | 198 | | 199 | 199 | 76 | 199 | 85 | 199 |  |  |  |
| Self Convict Crime Type | Pearson Correlation | -.102 | -.333 | .^c^ | -.426 | -.268 | .^c^ | -.079 | -.335 | .198 | -.281 | -.111 | -.111 | | .111 | .333 | 1.000^**^ | .272 | .316 | .^c^ | -- |  |  |
|  | Sig. (2-tailed) | .780 | .347 | . | .220 | .454 | . | .829 | .344 | .583 | .432 | .760 | .760 | | .760 | .347 | .000 | .447 | .541 | . |  |  |  |
|  | N | 10 | 10 | 9 | 10 | 10 | 10 | 10 | 10 | 10 | 10 | 10 | 10 | | 10 | 10 | 5 | 10 | 6 | 10 | 10 |  |  |
| Fam Convicted | Pearson Correlation | .061 | -.152^*^ | .097 | .022 | -.096 | .073 | -.024 | .120 | .151^*^ | .153^*^ | .056 | .140^*^ | | .366^**^ | .110 | .245^*^ | .256^**^ | .246^*^ | .384^**^ | .167 | -- |  |
|  | Sig. (2-tailed) | .395 | .033 | .180 | .758 | .178 | .305 | .742 | .090 | .034 | .031 | .431 | .050 | | <.001 | .121 | .033 | <.001 | .023 | <.001 | .645 |  |  |
|  | N | 197 | 196 | 192 | 199 | 199 | 198 | 199 | 199 | 199 | 199 | 199 | 198 | | 199 | 199 | 76 | 199 | 85 | 199 | 10 | 199 |  |
| Fam Convict Crime Type | Pearson Correlation | -.027 | .318 | -.271 | -.165 | -.033 | .^c^ | .092 | .012 | .249 | .101 | .000 | .131 | | -.134 | .171 | .214 | .326 | .456^*^ | -.210 | -.167 | .^c^ | -- |
|  | Sig. (2-tailed) | .884 | .071 | .140 | .359 | .853 | . | .611 | .945 | .163 | .575 | 1.000 | .466 | | .458 | .340 | .409 | .064 | .029 | .242 | .721 | . |  |
|  | N | 32 | 33 | 31 | 33 | 33 | 33 | 33 | 33 | 33 | 33 | 33 | 33 | | 33 | 33 | 17 | 33 | 23 | 33 | 7 | 33 | 33 |
| **. Correlation is significant at the 0.01 level (2-tailed). | | | | | | | | | | | | | | | | | | | | | | | |
| *. Correlation is significant at the 0.05 level (2-tailed). | | | | | | | | | | | | | | | | | | | | | | | |
| c. Correlation not reported because at least one of the variables is constant. | | | | | | | | | | | | | | | | | | | | | | | |

**Table 7.** Rehabilitation condition correlations with sentencing scores and individual difference variables

| **Correlations** | | | | | | | | | | | | | | | | | | | | | | |
| --- | --- | --- | --- | --- | --- | --- | --- | --- | --- | --- | --- | --- | --- | --- | --- | --- | --- | --- | --- | --- | --- | --- |
|  | | Sentencing Difference Scores | Gender | Race | Age | Schooling | Citizen | Political Ideology | Antisocial Scale Drug Abuse | Antisocial Scale Aggression | Antisocial Scale Total Score | Jury Service | Law Occupation | Arrested | Self Victim Crime | Self Type of Crime | Fam Victim Crime | Fam Type of Crime | Self Convicted | Self Convict Crime Type | Fam Convicted | Fam Convict Crime Type |
| Sentencing Difference Scores | Pearson Correlation | -- |  |  |  |  |  |  |  |  |  |  |  |  |  |  |  |  |  |  |  |  |
|  | N | 198 |  |  |  |  |  |  |  |  |  |  |  |  |  |  |  |  |  |  |  |  |
| Gender | Pearson Correlation | .015 | -- |  |  |  |  |  |  |  |  |  |  |  |  |  |  |  |  |  |  |  |
|  | Sig. (2-tailed) | .831 |  |  |  |  |  |  |  |  |  |  |  |  |  |  |  |  |  |  |  |  |
|  | N | 196 | 197 |  |  |  |  |  |  |  |  |  |  |  |  |  |  |  |  |  |  |  |
| Race | Pearson Correlation | .190^**^ | -.033 | -- |  |  |  |  |  |  |  |  |  |  |  |  |  |  |  |  |  |  |
|  | Sig. (2-tailed) | .009 | .652 |  |  |  |  |  |  |  |  |  |  |  |  |  |  |  |  |  |  |  |
|  | N | 189 | 190 | 190 |  |  |  |  |  |  |  |  |  |  |  |  |  |  |  |  |  |  |
| Age | Pearson Correlation | .277^**^ | .062 | .320^**^ | -- |  |  |  |  |  |  |  |  |  |  |  |  |  |  |  |  |  |
|  | Sig. (2-tailed) | <.001 | .387 | <.001 |  |  |  |  |  |  |  |  |  |  |  |  |  |  |  |  |  |  |
|  | N | 198 | 197 | 190 | 199 |  |  |  |  |  |  |  |  |  |  |  |  |  |  |  |  |  |
| Schooling | Pearson Correlation | -.031 | .153^*^ | -.023 | -.003 | -- |  |  |  |  |  |  |  |  |  |  |  |  |  |  |  |  |
|  | Sig. (2-tailed) | .662 | .032 | .750 | .961 |  |  |  |  |  |  |  |  |  |  |  |  |  |  |  |  |  |
|  | N | 198 | 197 | 190 | 199 | 199 |  |  |  |  |  |  |  |  |  |  |  |  |  |  |  |  |
| Citizen | Pearson Correlation | .115 | .007 | .153^*^ | .021 | -.075 | -- |  |  |  |  |  |  |  |  |  |  |  |  |  |  |  |
|  | Sig. (2-tailed) | .106 | .926 | .035 | .770 | .295 |  |  |  |  |  |  |  |  |  |  |  |  |  |  |  |  |
|  | N | 198 | 197 | 190 | 199 | 199 | 199 |  |  |  |  |  |  |  |  |  |  |  |  |  |  |  |
| Political Ideology | Pearson Correlation | .012 | -.103 | -.006 | -.114 | .174^*^ | .108 | -- |  |  |  |  |  |  |  |  |  |  |  |  |  |  |
|  | Sig. (2-tailed) | .867 | .150 | .932 | .110 | .014 | .132 |  |  |  |  |  |  |  |  |  |  |  |  |  |  |  |
|  | N | 197 | 196 | 189 | 198 | 198 | 198 | 198 |  |  |  |  |  |  |  |  |  |  |  |  |  |  |
| Antisocial Scale – Drug Abuse | Pearson Correlation | .088 | .073 | .187^**^ | .074 | -.249^**^ | .042 | .040 | -- |  |  |  |  |  |  |  |  |  |  |  |  |  |
|  | Sig. (2-tailed) | .217 | .309 | .010 | .296 | <.001 | .558 | .579 |  |  |  |  |  |  |  |  |  |  |  |  |  |  |
|  | N | 198 | 197 | 190 | 199 | 199 | 199 | 198 | 199 |  |  |  |  |  |  |  |  |  |  |  |  |  |
| Antisocial Scale – Aggression | Pearson Correlation | .002 | .253^**^ | -.133 | .056 | -.102 | .106 | -.014 | .345^**^ | -- |  |  |  |  |  |  |  |  |  |  |  |  |
|  | Sig. (2-tailed) | .977 | <.001 | .067 | .431 | .150 | .137 | .841 | <.001 |  |  |  |  |  |  |  |  |  |  |  |  |  |
|  | N | 198 | 197 | 190 | 199 | 199 | 199 | 198 | 199 | 199 |  |  |  |  |  |  |  |  |  |  |  |  |
| Antisocial Scale – Total Score | Pearson Correlation | .079 | .124 | .136 | .080 | -.247^**^ | .062 | .032 | .976^**^ | .540^**^ | -- |  |  |  |  |  |  |  |  |  |  |  |
|  | Sig. (2-tailed) | .266 | .083 | .061 | .263 | <.001 | .385 | .652 | <.001 | <.001 |  |  |  |  |  |  |  |  |  |  |  |  |
|  | N | 198 | 197 | 190 | 199 | 199 | 199 | 198 | 199 | 199 | 199 |  |  |  |  |  |  |  |  |  |  |  |
| Jury Service | Pearson Correlation | -.002 | -.008 | .034 | .277^**^ | .015 | .051 | .043 | .012 | .067 | .027 | -- |  |  |  |  |  |  |  |  |  |  |
|  | Sig. (2-tailed) | .976 | .910 | .642 | <.001 | .830 | .472 | .549 | .863 | .344 | .709 |  |  |  |  |  |  |  |  |  |  |  |
|  | N | 198 | 197 | 190 | 199 | 199 | 199 | 198 | 199 | 199 | 199 | 199 |  |  |  |  |  |  |  |  |  |  |
| Law Occupation | Pearson Correlation | -.025 | .086 | -.092 | -.015 | .077 | .016 | -.008 | .012 | .147^*^ | .045 | .156^*^ | -- |  |  |  |  |  |  |  |  |  |
|  | Sig. (2-tailed) | .729 | .228 | .209 | .836 | .280 | .821 | .910 | .866 | .038 | .530 | .027 |  |  |  |  |  |  |  |  |  |  |
|  | N | 198 | 197 | 190 | 199 | 199 | 199 | 198 | 199 | 199 | 199 | 199 | 199 |  |  |  |  |  |  |  |  |  |
| Arrested | Pearson Correlation | .079 | .073 | .105 | .194^**^ | -.225^**^ | .042 | -.123 | .625^**^ | .321^**^ | .635^**^ | .028 | .022 | -- |  |  |  |  |  |  |  |  |
|  | Sig. (2-tailed) | .267 | .308 | .151 | .006 | .001 | .552 | .083 | <.001 | <.001 | <.001 | .690 | .757 |  |  |  |  |  |  |  |  |  |
|  | N | 198 | 197 | 190 | 199 | 199 | 199 | 198 | 199 | 199 | 199 | 199 | 199 | 199 |  |  |  |  |  |  |  |  |
| Self Victim Crime | Pearson Correlation | .109 | -.006 | -.007 | .138 | -.112 | -.014 | -.044 | .281^**^ | .273^**^ | .315^**^ | -.018 | -.010 | .341^**^ | -- |  |  |  |  |  |  |  |
|  | Sig. (2-tailed) | .126 | .930 | .921 | .052 | .114 | .847 | .540 | <.001 | <.001 | <.001 | .801 | .884 | <.001 |  |  |  |  |  |  |  |  |
|  | N | 198 | 197 | 190 | 199 | 199 | 199 | 198 | 199 | 199 | 199 | 199 | 199 | 199 | 199 |  |  |  |  |  |  |  |
| Self Type of Crime | Pearson Correlation | .109 | -.361^**^ | -.131 | .089 | -.164 | -.128 | -.121 | .010 | .073 | .023 | .230^*^ | .025 | -.024 | .^c^ | -- |  |  |  |  |  |  |
|  | Sig. (2-tailed) | .322 | <.001 | .248 | .418 | .131 | .241 | .270 | .929 | .502 | .831 | .033 | .816 | .825 | . |  |  |  |  |  |  |  |
|  | N | 85 | 85 | 80 | 86 | 86 | 86 | 85 | 86 | 86 | 86 | 86 | 86 | 86 | 86 | 86 |  |  |  |  |  |  |
| Fam Victim Crime | Pearson Correlation | .156^*^ | .086 | -.019 | .102 | -.080 | -.101 | -.044 | .166^*^ | .318^**^ | .222^**^ | -.035 | .033 | .171^*^ | .532^**^ | -.052 | -- |  |  |  |  |  |
|  | Sig. (2-tailed) | .028 | .229 | .799 | .152 | .260 | .155 | .537 | .019 | <.001 | .002 | .626 | .644 | .016 | <.001 | .632 |  |  |  |  |  |  |
|  | N | 198 | 197 | 190 | 199 | 199 | 199 | 198 | 199 | 199 | 199 | 199 | 199 | 199 | 199 | 86 | 199 |  |  |  |  |  |
| FamTypeofCrime | Pearson Correlation | .038 | -.297^**^ | -.040 | .096 | .102 | -.167 | .012 | -.129 | -.167 | -.150 | .256^*^ | -.033 | -.145 | -.057 | .579^**^ | .^c^ | -- |  |  |  |  |
|  | Sig. (2-tailed) | .712 | .003 | .703 | .344 | .317 | .098 | .905 | .201 | .099 | .139 | .010 | .749 | .151 | .578 | <.001 | . |  |  |  |  |  |
|  | N | 98 | 99 | 94 | 99 | 99 | 99 | 98 | 99 | 99 | 99 | 99 | 99 | 99 | 99 | 69 | 99 | 99 |  |  |  |  |
| Self Convicted | Pearson Correlation | .024 | .077 | .042 | -.044 | -.150^*^ | .023 | -.062 | .577^**^ | .286^**^ | .583^**^ | -.003 | .110 | .482^**^ | .171^*^ | -.028 | .139^*^ | -.180 | -- |  |  |  |
|  | Sig. (2-tailed) | .742 | .280 | .565 | .535 | .035 | .745 | .387 | <.001 | <.001 | <.001 | .962 | .122 | <.001 | .016 | .796 | .050 | .075 |  |  |  |  |
|  | N | 198 | 197 | 190 | 199 | 199 | 199 | 198 | 199 | 199 | 199 | 199 | 199 | 199 | 199 | 86 | 199 | 99 | 199 |  |  |  |
| Self Convict Crime Type | Pearson Correlation | .^c^ | .^c^ | .^c^ | .^c^ | .^c^ | .^c^ | .^c^ | .^c^ | .^c^ | .^c^ | .^c^ | .^c^ | .^c^ | .^c^ | .^c^ | .^c^ | .^c^ | .^c^ | .^c^ |  |  |
|  | Sig. (2-tailed) | . | . | . | . | . | . | . | . | . | . | . | . | . | . | . | . | . | . |  |  |  |
|  | N | 10 | 10 | 9 | 10 | 10 | 10 | 10 | 10 | 10 | 10 | 10 | 10 | 10 | 10 | 8 | 10 | 8 | 10 | 10 |  |  |
| Fam Convicted | Pearson Correlation | -.014 | -.084 | -.063 | -.045 | -.175^*^ | .044 | -.120 | .295^**^ | .123 | .292^**^ | .149^*^ | .105 | .236^**^ | .088 | .111 | .166^*^ | .033 | .275^**^ | .^c^ | -- |  |
|  | Sig. (2-tailed) | .840 | .239 | .391 | .525 | .013 | .536 | .093 | <.001 | .084 | <.001 | .036 | .142 | <.001 | .219 | .307 | .019 | .747 | <.001 | . |  |  |
|  | N | 198 | 197 | 190 | 199 | 199 | 199 | 198 | 199 | 199 | 199 | 199 | 199 | 199 | 199 | 86 | 199 | 99 | 199 | 10 | 199 |  |
| Fam Convict Crime Type | Pearson Correlation | .079 | -.010 | -.309 | .074 | .139 | .^c^ | .068 | -.005 | .201 | .034 | .094 | .176 | .094 | .346 | .450 | .194 | .466^*^ | -.061 | .^c^ | .^c^ | -- |
|  | Sig. (2-tailed) | .667 | .959 | .103 | .687 | .447 | . | .710 | .978 | .269 | .854 | .607 | .336 | .607 | .053 | .070 | .288 | .029 | .742 | . | . |  |
|  | N | 32 | 32 | 29 | 32 | 32 | 32 | 32 | 32 | 32 | 32 | 32 | 32 | 32 | 32 | 17 | 32 | 22 | 32 | 6 | 32 | 32 |
| **. Correlation is significant at the 0.01 level (2-tailed). | | | | | | | | | | | | | | | | | | | | | | |
| *. Correlation is significant at the 0.05 level (2-tailed). | | | | | | | | | | | | | | | | | | | | | | |
| c. Correlation not reported because at least one of the variables is constant. | | | | | | | | | | | | | | | | | | | | | | |

**Control condition.** In the control condition, participants with family members who had been convicted of a violent crime (*n* = 11) were less likely to reduce their sentencing scores from pretest to post-test, *r*(38) = .35, *p* = .026 (see Table 1). In addition, there was a positive correlation between U.S. citizenship status and sentencing difference scores, *r*(182) = .22, *p* = .003, however only 1 participant indicated that they were not a U.S. citizen in this condition, so we did not examine this correlation further.

**Table 1.** Mean sentencing scores of participants who had a family member convicted of a crime, broken down by crime type

|  | **Pretest score** | **Post-test score** |
| --- | --- | --- |
| **Non-violent crime** | *M* = 13.48, *SD* = 4.65  *n* = 29 | *M* = 12.69, *SD* = 4.91  *n* = 29 |
| **Violent crime** | *M* = 12.45, *SD* = 3.47  *n* = 11 | *M* = 13.55, *SD* = 4.30  *n* = 11 |

**Retribution condition.** In the Retribution condition, compared to participants with no convicted family member, participants who had a family member convicted of a crime (*n* = 36) showed a greater reduction in their sentencing judgements from pretest to post-test when the prison rationale was retribution, *r*(196) = -.23, *p* < .001 (see Table 2).

**Table 2.** Mean sentencing scores, broken down by participants who did and did not have a family member convicted of a crime

|  | **Pretest score** | **Post-test score** |
| --- | --- | --- |
| **No convicted family member** | *M* = 13.02, *SD* = 4.36  *n* = 162 | *M* = 12.71, *SD* = 4.52  *n* = 163 |
| **Convicted family member** | *M* = 13.19, *SD* = 4.58  *n* = 37 | *M* = 11.81, *SD* = 4.85  *n* = 36 |

**Public safety condition.** Among participants in the public safety condition, older participants were more likely to increase their post-test sentencing recommendations in this condition, *r*(195) = .15, *p* = .032. Female participants also were more likely to increase sentences, *r*(192) = -.20, *p* = .005 (see Table 3). In addition, participants with more education were also more likely to increase their post-test sentencing scores, *r*(195) = .20, *p* = .005.

**Table 3.** Mean sentencing scores, broken down by gender

|  | **Pretest score** | **Post-test score** |
| --- | --- | --- |
| **Female** | *M* = 12.67, *SD* = 4.34  *n* = 98 | *M* = 14.10, *SD* = 4.65  *n* = 98 |
| **Male** | *M* = 13.65, *SD* = 4.65  *n* = 96 | *M* = 13.95, *SD* = 4.91  *n* = 96 |

Note: gender was recoded to be a binary variable (i.e., female vs. male). Participants who endorsed a non-binary identity or who did not report their gender were excluded from these analyses.

**Rehabilitation condition.** In the rehabilitation condition, older participants were more likely to increase their post-test sentencing recommendations, *r*(196) = .28, *p* < .001. In addition, participants who had a family member who was a victim of a crime (*n* = 99) were more likely to increase their sentencing scores from pretest to post-test, *r*(196) = .16, *p* = .028 (see Table 4). Participants who identified as white were also more likely to increase their post-test sentences, *r*(187) = .19, *p* = .009 (see Table 5).

**Table 4.** Mean sentencing scores, broken down by participants who did and did not have a family member who had been the victim of a crime

|  | **Pretest score** | **Post-test score** |
| --- | --- | --- |
| **No crime victim family member** | *M* = 12.18, *SD* = 4.36  *n* = 100 | *M* = 12.38, *SD* = 4.70  *n* = 100 |
| **Crime victim family member** | *M* = 12.11, *SD* = 4.17  *n* = 98 | *M* = 13.08, *SD* = 4.78  *n* = 99 |

**Table 5.** Mean sentencing scores, broken down by race

|  | **Pretest score** | **Post-test score** |
| --- | --- | --- |
| **Non-white** | *M* = 12.18, *SD* = 4.37  *n* = 34 | *M* = 11.68, *SD* = 4.37  *n* = 34 |
| **White** | *M* = 12.08, *SD* = 4.22  *n* = 155 | *M* = 12.96, *SD* = 4.79  *n* = 155 |

Note: race was recoded to be a binary variable (i.e., non-white vs. white).

**Robustness with age as a covariate.** Given that age correlated with difference scores in two of the conditions, the omnibus analyses reported in the main section (i.e., condition by timepoint) were re-run out with age as a covariate. There was still a significant interaction effect: *F*(3, 773) = 21.26, *p* < .001, η^2^*_p_* = .08. There also was a significant main effect of time, *F*(1, 773) = 14.98, *p* < .001, *d*_z_ = .27, with higher scores overall at post-test (*M* = 13.17, *SD* = .17) than at pretest (*M* = 13.13, *SD* = .16) and a significant main effect of condition, *F*(3, 773) = 4.05, *p* = .007, η^2^*_p_* = .015.

**3c. One-way ANOVA examining effect of conditions on baseline sentencing judgements**

The pretest scores were measured after participants were told about what policy the hypothetical country holds. Thus, the baseline ratings provide additional data on how people’s sentencing judgments vary as a function of the goal of imprisonment. A one-way ANOVA testing the effect of the conditions on participants’ baseline sentencing judgements was significant, *F*(3, 777) = 6.26, *p* < .001, η^2^*_p_* = .024. Pairwise comparisons using a Bonferroni correction showed that this effect was because people who thought about the rehabilitation rationale recommended significantly shorter sentences at baseline (*M* = 12.15), compared to control condition participants (*M* = 14.11). This is understandable because participants assigned to the rehabilitation condition were explicitly instructed to consider a more humane approach to the criminal legal system when recommending a sentence. They may have associated this more humane approach with shorter sentences at baseline.

**4. Balance test**

To ensure that participant characteristics did not vary significantly across our conditions, we conducted a balance test using gender, age, education level and political orientation. A chi-square test revealed no significant relation between participant gender and condition assignment, *X^2^* (3, *N* = 776) = 6.20, *p* =.102. A one-way ANOVA revealed no significant effect of condition on age, *F*(3, 780) = 1.24, *p* = .294, η^2^*_p_* = .005, or on education level, *F*(3, 780) = .73, *p* = .534, η^2^*_p_* = .003, or on political orientation, *F*(3, 779) = .73, *p* = .536, η^2^*_p_* = .003. In addition, our attention checks and education requirement excluded participants across all conditions (*n* = 4 in the retribution condition, *n* = 4 in the rehabilitation condition, *n* = 3 in the public safety condition, and *n* = 7 in the control condition).

**References**

**1.** Aspinwall LG, Brown TR, Tabery J. The double-edged sword: Does biomechanism increase or decrease judges' sentencing of psychopaths?. Science. 2012;337(6096):846-9.

**2.** Orlando, J. Crimes with Mandatory Minimum Prison Sentences — Updated and Revised. Office of Legislative Research. 2017.

**3.** Remmel RJ, Glenn AL, Cox J. Biological evidence regarding psychopathy does not affect mock jury sentencing. Journal of Personality Disorders. 2019;33(2):164-84.
